# Solution of Turbulent Flow and Solidification in Continuous Casting of Round Steel Billets with a Meshless Method

**DOI:** 10.3390/ma19204428

**Published:** 2026-10-21

**Authors:** Katarina Mramor, Zlatko Rek, Robert Vertnik, Božidar Šarler

**Affiliations:** 1 Faculty of Mechanical Engineering, University of Ljubljana, Aškerčeva cesta 6, SI-1000 Ljubljana, Slovenia; katarina.mramor@fs.uni-lj.si (K.M.); zlatko.rek@guest.arnes.si (Z.R.); 2 Štore Steel, d.o.o., Železarska cesta 3, SI-3220 Štore, Slovenia; robert.vertnik@store-steel.si

**Keywords:** continuous casting of steel, meshless numerical method, radial basis function, solidification, turbulence, axisymmetry, mixture continuum model

## Abstract

Continuous casting is the most widely used process in the steelmaking industry. Understanding the physical phenomena and modeling them is essential for mitigating casting defects. This study uses a mixture-continuum formulation in axisymmetry to model this process computationally. A physical model incorporating coupled mass, momentum, and energy equations for solidification with melt turbulence was employed, assuming Newtonian, incompressible behavior and the k-epsilon turbulence model. The model provides insights into the thermo-fluid dynamics during solidification. For the numerical simulation, the Local Radial Basis Function Collocation Method (LRBFCM), a meshless approach, is utilized and compared against the more traditional Finite Volume Method (FVM). The results demonstrate that the meshless LRBFCM offers comparable accuracy and convergence to the FVM while reducing the computational complexity associated with mesh generation. These findings suggest that LRBFCM is a viable alternative for simulating complex metallurgical processes, such as continuous casting, with competitive performance.

## 1. Introduction

In the metallurgical industry, the continuous casting (CC) process is the most widely used approach for producing crude steel semi-finished products, accounting for over 96.5% of steel produced [1] in 2024. The products can be cast in various shapes and sizes, ranging from round and square billets to blooms and slabs. The quality of the cast product largely depends on controlling the complex physical phenomena of the process. High temperatures, inaccessibility of the mold region, large-scale casting, and the high cost of setting up process parameters for new steel grades and formats by trial and error are reasons for the increased use of numerical simulations. In recent years, advances in computational power have enabled successful simulations of three-dimensional (3D) CC problems [2,3,4,5,6,7,8]. The 3D simulations most accurately model the actual process but are still excessively time-consuming for optimization, which requires many subsequent runs. The 3D numerical models can be reduced to an axisymmetric formulation for the billet’s round geometry and boundary conditions, which is the objective of this paper. Axisymmetric models in metal casting have been addressed using various numerical methods, ranging from the classical finite volume method (FVM) [9] and finite difference method (FDM) [10] to contemporary meshless methods [11].

The meshless Local Radial Basis Function Collocation Method (LRBFCM) [12] is used in the present paper to solve coupled partial differential equations that mathematically describe the temperature and turbulent velocity field in the continuous casting process. The problem’s equations are devised using a mixture continuum model that encompasses mass, momentum, and energy conservation equations for the solidification process. The turbulent nature of the flow is approximated by the Reynolds-Averaged Navier–Stokes (RANS) model, which provides a steady-state time-averaged flow solution. More sophisticated turbulence simulation methods, such as large eddy simulation (LES) or even direct numerical simulation (DNS), can capture the transient nature of turbulence and thus provide more accurate results. The choice of turbulence model significantly affects both computational complexity and the final calculated solidification profile [13,14]. However, the LES and DNS models are computationally costly and impractical for most industrial applications. Since we adopt the incompressibility approximation for solidifying steel, the fractional step method [15] can reliably and accurately handle the coupling between pressure and velocity.

The latter provides a promising alternative approach for macroscopic modeling of solidification. The Finite Point Method (FPM) [16,17,18] and Element Free Galerkin [19,20,21] methods have been applied to modeling transient heat transfer, enthalpy, shell growth, and thermo-mechanical calculations. In contrast, the Meshless Local Petrov–Galerkin method coupled with FPM [17] is highly applicable to thermo-mechanical and stress analysis problems. The smoothed particle hydrodynamics [22,23] and meshless RBF-cellular and point automata [24] approaches can address free-surface and multiphase transitions and have been used to predict macroscopic temperature fields, as well as columnar-to-equiaxed grain evolution. More recently, the Material Point Method [25,26] provides a mesh-independent framework for coupled thermal, mechanical, and phase-change problems, while Optimal Transportation methods [27,28] provide a particle-based formulation in which the computational points and their transport can be determined through optimal mass redistribution. Collectively, these approaches offer different levels of capability, from macroscopic temperature and shell-thickness prediction to fully coupled thermo-fluid, thermo-mechanical, and microstructural simulations of continuous casting.

In particular, LRBFCM provides high-order spatial approximation and good numerical accuracy through RBF interpolation while retaining a local formulation, thereby reducing computational cost and memory requirements compared with global RBF approaches. Its local support also allows discretization to be adapted to non-uniform node distributions, which is advantageous for continuous casting problems involving strong gradients in the mushy and boundary regions. Furthermore, because LRBFCM does not require a fixed mesh connectivity, local node refinement can be introduced in regions of particular interest without remeshing the entire computational domain. Boundary conditions can be imposed directly at boundary nodes through the collocation formulation, avoiding some of the additional boundary-treatment procedures commonly required in particle-based methods such as SPH. In comparison, SPH provides a highly flexible particle-based framework and naturally accommodates large deformation and free-surface motion, but may require special treatments near boundaries and can suffer from reduced accuracy or particle-distribution-related errors. MPM is particularly advantageous for problems involving large deformation and complex solid mechanics, but its computational framework is more complex and generally requires treating particles alongside a background grid. OTM and related particle-based approaches similarly provide flexibility in node/particle distribution but may require additional stabilization or special boundary treatments. The limitations of LRBFCM include the sensitivity to node distribution, support-node selection, RBF shape parameters, and matrix conditioning. Nevertheless, in the present continuous casting problem, where accurate resolution of thermal and velocity gradients, flexible node placement, and efficient local discretization are important, these characteristics provide a strong motivation for using LRBFCM. The present contribution aims to extend [11] and to evaluate the convergence of various numerical methods and algorithms, while also generating an axisymmetric reference numerical solution for future comparisons. The extension considers two computational domains instead of one; a detailed configuration including a submerged entry nozzle (SEN) and a simplified configuration without it. This allows for a clearer assessment of the influence of geometric complexity on the predicted flow and temperature fields. In addition, the present study investigates the influence of node arrangement using a wider range of nodal distributions, providing a more systematic evaluation of the effects of spatial discretization than that presented in [11]. Building upon the framework presented in article [11], this extension formulates the governing equations explicitly in the r and z directions, thereby providing a direction-specific representation rather than the generalized vector formulation used in the original work. A more comprehensive analysis of temporal discretization is also performed, including an investigation of the influence of the time-step size on the stability and accuracy of the simulations. Furthermore, the present work provides a detailed assessment of the stability criteria relevant to the LRBFCM formulation, with particular attention to the stability criteria of diffusion and convection terms and their implications for the simulation. Finally, the present study reports and discusses computational times for different cases, thereby enabling assessment of the method’s computational efficiency in addition to its accuracy and stability. 

## 2. Governing Equations

A domain Ω with boundary Γ is occupied by metal undergoing a phase change from liquid ℘=L to solid ℘=S phase in an axisymmetric coordinate system, i.e., point x=(r,z) with unit base vectors ${\overset{ˆ}{i}}_{\zeta };\zeta =r,\;z$. The equation of Newtonian axisymmetric incompressible turbulent solidifying flow is governed by macroscopic conservation equations for mass, momentum, energy [29], turbulent kinetic energy, and dissipation rate [30] formulated in a mixture continuum formulation

(1) $$\frac{1}{r}\frac{\partial (ru_{r})}{\partial r}+\frac{\partial u_{z}}{\partial z}=0$$

(2) $$\begin{array}{c} \rho \frac{\partial u_{r}}{\partial t}+\rho \left(u_{r}\frac{\partial u_{r}}{\partial r}+u_{z}\frac{\partial u_{r}}{\partial z}\right)=-\frac{\partial p}{\partial r}+\left(\mu_{L}+\mu_{t}\right)\left(\frac{1}{r}\frac{\partial }{\partial r}\left(r\frac{\partial u_{r}}{\partial r}\right)+\frac{{\partial^{2}u}_{r}}{\partial z^{2}}-\frac{u_{r}}{r^{2}}\right)\\ -\frac{2}{3}\rho \frac{\partial k}{\partial r}-\mu_{L}\frac{K_{0}{\left(1-f_{L}\right)}^{2}}{f_{L}^{3}}\left(u_{r}-u_{r,S}\right) \end{array}$$

(3) $$\begin{array}{c} \rho \frac{\partial u_{z}}{\partial t}+\rho \left(u_{r}\frac{\partial u_{z}}{\partial r}+u_{z}\frac{\partial u_{z}}{\partial z}\right)=-\frac{\partial p}{\partial z}+\left(\mu_{L}+\mu_{t}\right)\left(\frac{1}{r}\frac{\partial }{\partial r}\left(r\frac{\partial u_{z}}{\partial r}\right)+\frac{{\partial^{2}u}_{z}}{\partial z^{2}}\right)\\ -\frac{2}{3}\rho \frac{\partial k}{\partial z}-\mu_{L}\frac{K_{0}{\left(1-f_{L}\right)}^{2}}{f_{L}^{3}}\left(u_{z}-u_{z,S}\right)+\rho \beta_{T}g_{z}\left(T-T_{ref}\right) \end{array}$$

(4) $$\begin{array}{c} \rho \frac{\partial h}{\partial t}+\rho \left(\frac{\partial h}{\partial r}u_{r}+\frac{\partial h}{\partial z}u_{z}\right)=\lambda \left(\frac{1}{r}\frac{\partial }{\partial r}\left(r\frac{\partial T}{\partial r}\right)+\frac{\partial^{2}T}{\partial z^{2}}\right)+\left(f_{L}\frac{\mu_{t}}{\sigma_{t}}\right)\left(\frac{1}{r}\frac{\partial }{\partial r}\left(r\frac{\partial h_{L}}{\partial r}\right)+\frac{\partial^{2}h_{L}}{\partial z^{2}}\right)\\ +\rho [\left(1-2f_{L}\right)\left(h_{S}-h_{L}\right)\left(\left(u_{r,L}-u_{r,S}\right)\frac{\partial f_{L}}{\partial r}+\left(u_{z,L}-u_{z,S}\right)\frac{\partial f_{L}}{\partial z}\right)\\ +f_{L}\left(1-f_{L}\right)\left(h_{S}-h_{L}\right)\left(\frac{1}{r}\frac{\partial }{\partial r}\left(r\left(u_{r,L}-u_{r,S}\right)\right)+\frac{\partial \left(u_{z,L}-u_{z,S}\right)}{\partial z}\right)\\ +f_{L}\left(1-f_{L}\right)\left(\frac{\partial \left(h_{S}-h_{L}\right)}{\partial r}\left(u_{r,L}-u_{r,S}\right)+\frac{\partial \left(h_{S}-h_{L}\right)}{\partial z}\left(u_{z,L}-u_{z,S}\right)\right)] \end{array}$$

(5) $$\begin{array}{c} \rho \frac{\partial \varepsilon }{\partial t}+\rho \left(\frac{\partial \varepsilon }{\partial r}u_{r}+\frac{\partial \varepsilon }{\partial z}u_{z}\right)=\left(\mu_{L}+\frac{\mu_{t}}{\sigma_{\varepsilon }}\right)\left(\frac{1}{r}\frac{\partial }{\partial r}\left(r\frac{\partial \varepsilon }{\partial r}\right)+\frac{\partial^{2}\varepsilon }{\partial z^{2}}\right)+\rho E\\ -\mu_{L}\frac{K_{0}{\left(1-f_{L}\right)}^{2}}{f_{L}^{3}}\varepsilon +\left[c_{1\varepsilon }f_{1}\left(P_{k}+c_{3\varepsilon }G_{k}\right)-c_{2\varepsilon }f_{2}\rho \varepsilon \right]\frac{\varepsilon }{k} \end{array}$$

(6) $$\begin{array}{c} \rho \frac{\partial k}{\partial t}+\rho \left(\frac{\partial k}{\partial r}u_{r}+\frac{\partial k}{\partial z}u_{z}\right)=\left(\mu_{L}+\frac{\mu_{t}}{\sigma_{k}}\right)\left(\frac{1}{r}\frac{\partial }{\partial r}\left(r\frac{\partial k}{\partial r}\right)+\frac{\partial^{2}k}{\partial z^{2}}\right)+\\ -\mu_{L}\frac{K_{0}{\left(1-f_{L}\right)}^{2}}{f_{L}^{3}}k+P_{k}+G_{k}-\rho \varepsilon +-\rho D \end{array}$$

where symbols stand for density (ρ), time (t), mixture velocity $\left(u_{\zeta }\right)$, pressure (p), dynamic viscosity of liquid ($\mu_{L}$), turbulent dynamic viscosity ($\mu_{t}$), turbulent kinetic energy (k), volume fraction of phase $℘\left(f_{℘}\right)$, permeability constant $\left(K_{0}\right)$, velocity of phase $℘\left(u_{\zeta ,℘}\right)$, thermal expansion coefficient $\left(\beta_{T}\right)$, gravitational acceleration $\left(g_{z}\right)$, temperature (T), reference temperature ($T_{\mathrm{ref}\;}$), mixture enthalpy (h), thermal conductivity (λ), turbulent Prandtl number ($\sigma_{t}$), enthalpy of phase $℘\left(h_{℘}\right)$, turbulent closure coefficient for $k\left(\sigma_{k}\right)$, shear production of turbulent kinetic energy $\left(P_{k}\right)$, generation of turbulence due to the buoyancy force $\left(G_{k}\right)$, dissipation rate (ε), source of k (D), turbulent closure coefficient for $\varepsilon \;\left(\sigma_{\varepsilon }\right)$, source of ε(E), closure coefficients ($c_{1\varepsilon },f_{1},c_{3\varepsilon }$), and damping functions ($c_{2\varepsilon },f_{2}$) of low-Re k-ε turbulence model. Mixture velocity $u_{\zeta }=f_{S}u_{\zeta ,S}+f_{L}u_{\zeta ,L}$ is a linear combination of phase velocities $u_{\zeta ,℘}$ and phase volume fractions $f_{℘}$ with constraint $f_{S}+f_{L}=1$. In the fully liquid region ($f_{L}=1$), the mixture velocity reduces to the liquid velocity, whereas in the fully solid region ($f_{S}=1$), the mixture velocity corresponds to the prescribed casting/solid velocity. In the present model, the velocity of the fully solidified phase is prescribed to be equal to the casting velocity ($u_{\mathrm{cast}\;}$). This assumption represents the physical condition in which the solidified material moves together with the continuously withdrawn casting. The interaction between the solid and liquid phases in the mushy zone is accounted for implicitly through the mixture formulation and phase fractions, rather than by solving separate momentum equations for each phase. This approach is appropriate for the present macroscopic description of the solidification process, where the primary objective is to predict the flow and thermal behavior during solidification.

Mixture enthalpy $h=f_{S}h_{S}+f_{L}h_{L}$ is in turn a linear combination of phase volume fractions $f_{℘}$ and phase enthalpies $h_{℘}$, which are coupled with temperature through the following temperature-enthalpy constitutive relations

(7) $$h_{S}=\int_{T_{ref}}^{T}c_{pS}dT,h_{L}=\int_{T_{ref}}^{T_{S}}c_{pS}dT+\int_{T_{S}}^{T}c_{pL}dT+h_{m}$$

where $c_{p_{℘}}$ is the specific heat of the phase ℘ and $h_{m}$ is the melting enthalpy. The coupling between temperature and phase volume fraction is done through the lever microsegregation rule

(8) $$f_{L}=\{\begin{array}{ll} 1 & ;T>T_{L}\\ 1-\frac{T_{L}-T}{T_{L}-T_{S}} & ;T_{L}\geq T\geq T_{S}\\ 0 & ;T<T_{S} \end{array}$$

The shear production of turbulent kinetic energy is calculated as

(9) $$P_{k}=\mu_{t}\left[2\left({\left(\frac{1}{r}\frac{\partial \left(ru_{r}\right)}{\partial r}\right)}^{2}+{\left(\frac{\partial u_{z}}{\partial z}\right)}^{2}\right)+{\left(\frac{\partial u_{r}}{\partial z}+\frac{\partial u_{z}}{\partial r}\right)}^{2}\right]$$

and the generation of turbulence due to buoyancy as

(10) $$G_{k}=-g_{z}\beta_{T}\frac{\mu_{t}}{\sigma_{t}}\frac{\partial T}{\partial z}$$

The Abe–Kondoh–Nagano closure coefficients $(\sigma_{k}=1.50,\sigma_{\varepsilon }=1.90,c_{1\varepsilon }=$ 1.40, $c_{3\varepsilon }=tanh\left|u_{\zeta ,\|}/u_{\zeta ,⊥}\right|,f_{1}=1.0$), source terms (D=0,E=0) and damping functions $\left(c_{2\varepsilon }=1.40,f_{2}={\left[1-exp\left(-y*/3.1\right)\right]}^{2}\times \left[1-0.3exp{\left(-Re_{t}/6.5\right)}^{2}\right]\right)$ are used in connection with turbulent dynamic viscosity, defined as

(11) $$\mu_{t}=\rho c_{\mu }f_{\mu }\frac{k^{2}}{\varepsilon }$$

where $c_{\mu }=0.09$ is the closure coefficient and

(12) $$f_{\mu }={\left[1-exp\left(-y*/14\right)\right]}^{2}\times \left[1+\left(5/Re_{t}^{0.75}\right)exp{\left(-Re_{t}/200\right)}^{2}\right]$$

is the damping function of the turbulent model. $u_{\zeta ,\|}$ and $u_{\zeta ,⊥}$ are parallel and perpendicular velocities, $y*=\left(\rho u_{\varepsilon }r\right)/\mu$ is a non-dimensional distance with r representing radial distance from the billet’s outer wall, $u_{\varepsilon }=(\mu \varepsilon /\rho {)}^{0.25}$ representing the Kolmogorov velocity scale, and $Re_{t}=k^{2}\rho /(\mu \varepsilon )$ turbulent Reynolds number.

The solution of the above-mentioned governing equations is obtained for Reynolds time-averaged velocity, temperature, turbulent kinetic energy, and dissipation rate fields calculated by solving the equations for $t_{n+1}=t_{n}+\Delta t$, where $t_{n},n=0,\ldots ,N$ is the time step with $t_{0}$ representing the initial time, Δt time increment, and $t_{N}$ the final time, when the solution converges to a steady state.

The application of the mixture continuum model introduces several simplifying assumptions. The solid phase is treated as a rigid body that moves with the casting; therefore, solid deformation is neglected. Consequently, elastic or plastic deformation of the solidified material, as well as stresses generated within the solid shell, are not resolved. The model also neglects solidification shrinkage, so the density and volume changes associated with the transformation from liquid to solid are not explicitly coupled to the velocity field. Therefore, shrinkage-induced flow, deformation of the solid shell, and the associated changes in the geometry are not captured.

Furthermore, thermo-mechanical coupling is not considered explicitly. Temperature-dependent thermal expansion or contraction, thermal stresses, residual stresses, and the mechanical response of the solid shell are outside the scope of the present formulation. As a result, the model should be interpreted as a thermal–fluid-dynamic description of the solidification process rather than a fully coupled thermo-mechanical solidification model.

## 3. Numerical Method

### 3.1. Forward Euler Scheme

The time discretization is performed using the forward Euler scheme, a method known to be first-order accurate in time. The local truncation error per time step is thus proportional to the square of the time step size $\left(\Delta t^{2}\right)$, while the global error over the entire integration period is proportional to the step size. Therefore, the method’s overall accuracy improves linearly as the time step decreases.

### 3.2. Local Radial Basis Function Collocation Method

The space discretization is performed using LRBFCM and closely follows the work presented in our previous papers [2,12]. A general variable ϕ is a function of node position x in a local sub-domain, approximated as the sum of $N_{\mathrm{basis}\;}$ shape functions ψ and m monomials ϑ, both multiplied by shape coefficients α

(13) $$\phi (x)\approx \sum_{j=1}^{N_{\mathrm{basis}\;}}\psi_{j}(x)\alpha_{j}+\sum_{j=1}^{m}\vartheta_{j}(x)\alpha_{N_{\mathrm{basis}\;}+j}=\sum_{j}^{N_{\mathrm{basis}\;}+m}\Psi (x)\alpha_{j}$$

The general variable, which in our case represents both vector (velocity) and scalar fields (enthalpy, turbulent kinetic energy, dissipation rate), is defined on a local sub-domain _*l*_ with $N_{l}=N_{l\Omega }+N_{l\Gamma }$ grid-nodes, where $N_{l\Omega }$ are grid-nodes in the domain and $N_{l\Gamma }$ on the boundary.

Shape coefficients are calculated from the known nodal values in the sub-domain by collocation, which implies that

(14) ϕ(xnl)=∑jNbasis +mΨ(xnl)αj,

where ϕ(xnl)=ϕnl is the function value at the node xnl;n=1,…,Nl in sub-domain _*l*_. The system of equations is rewritten in matrix form as

(15) $$\begin{array}{l} \Psi \alpha =\Phi ;\\ \Psi =\left[\begin{array}{ccc} \psi_{1}\left(x_{1}\right) & \ldots & \begin{array}{ccc} \psi_{N_{\mathrm{basis}\;}}\left(x_{1}\right) & \vartheta_{1}\left(x_{1}\right) & \begin{array}{cc} \ldots & \vartheta_{m}\left(x_{1}\right) \end{array} \end{array}\\ \vdots & \ddots & \begin{array}{ccc} \vdots & \vdots & \begin{array}{cc} \ddots & \vdots \end{array} \end{array}\\ \begin{array}{c} \psi_{1}\left(x_{N_{\mathrm{basis}\;}}\right)\\ \vartheta_{1}\left(x_{N_{\mathrm{basis}\;}+1}\right)\\ \begin{array}{c} \vdots \\ \vartheta_{1}\left(x_{N_{\mathrm{basis}\;}+m}\right) \end{array} \end{array} & \begin{array}{c} \ldots \\ \ldots \\ \begin{array}{c} \ddots \\ \ldots \end{array} \end{array} & \begin{array}{c} \begin{array}{ccc} \psi_{N_{\mathrm{basis}\;}}\left(x_{N_{\mathrm{basis}\;}}\right) & \vartheta_{1}\left(x_{N_{\mathrm{basis}\;}}\right) & \begin{array}{cc} \ldots & \vartheta_{m}\left(x_{N_{\mathrm{basis}\;}}\right) \end{array} \end{array}\\ \begin{array}{ccc} \vartheta_{m}\left(x_{N_{\mathrm{basis}\;}+1}\right) & 0 & \begin{array}{cc} \ldots & 0 \end{array} \end{array}\\ \begin{array}{ccc} \begin{array}{c} \vdots \\ \vartheta_{m}\left(x_{N_{\mathrm{basis}\;}+m}\right) \end{array} & \begin{array}{c} \vdots \\ 0 \end{array} & \begin{array}{c} \begin{array}{cc} \ddots & \vdots \end{array}\\ \begin{array}{cc} \ldots & 0 \end{array} \end{array} \end{array} \end{array} \end{array}\right];\\ \alpha =\left[\begin{array}{c} \alpha_{1}\\ \vdots \\ \begin{array}{c} \alpha_{N_{\mathrm{basis}\;}}\\ \alpha_{N_{\mathrm{basis}+1}}\\ \begin{array}{c} \vdots \\ \alpha_{N_{\mathrm{basis}+m}} \end{array} \end{array} \end{array}\right];\;\Phi =\left[\begin{array}{c} \phi_{1}\\ \vdots \\ \begin{array}{c} \phi_{N_{\mathrm{basis}\;}}\\ 0\\ \begin{array}{c} \vdots \\ 0 \end{array} \end{array} \end{array}\right] \end{array}$$

where Ψ is the collocation matrix, α the matrix containing shape coefficients, and Φ the matrix containing function values. The computation of coefficients $\alpha =\Psi^{-1}\Phi$ is possible by inverting the system if the number of domain nodes coincides with the number of shape functions ($N_{\mathrm{basis}\;}=N_{l}$) and if the collocation matrix is non-singular.

In the present paper, the local sub-domain consists of five nodes. For each calculation node, four neighboring nodes are selected based on proximity, subject to the condition that each neighbor lies in a different spatial direction from the calculation node. The entire computational domain surrounding each calculation node is divided into four quarters. One node is then selected from each quarter, with the selected node being the closest available node to the calculation node within that quarter. This directional node selection strategy provides well-distributed local support. It avoids concentrating neighboring nodes in a single region, which can adversely affect the conditioning and numerical stability of the local RBF interpolation. The shape parameter is 32. The condition number is calculated with the dgecon () algorithm from the LAPACK library [31] as

(16) $$R_{\mathrm{cond}\;}=1/\left({\left||\Psi |\right|}_{1}{\left||\Psi^{-1}|\right|}_{1}\right).$$

The limit for the condition number is set to $10^{-10}$. Both the shape parameter and the limiting value for the condition number were selected based on the sensitivity analysis reported in our previous study [12].

### 3.3. Radial Basis Functions and Augmentation Monomials in Axisymmetry

The shape function utilizes multiquadric (MQ) radial basis functions (RBF), which are, in axisymmetry, defined as

(17) $$\psi_{j}(x)=\sqrt{x_{j}^{2}+c^{2}};\;\;x_{j}^{2}=\frac{{\left(r-r_{j}\right)}^{2}}{r_{j,max}^{2}}+\frac{{\left(z-z_{j}\right)}^{2}}{z_{j,max}^{2}}$$

with c standing for the shape parameter, $r_{j,max}$ for the maximum radial distance between two points in the sub-domain and $z_{j,max}$ the maximum distance between two points in the axial direction in the sub-domain. The derivatives of MQ RBFs in axisymmetry are

(18) $$\frac{\partial \psi_{j}}{\partial r}=\frac{r-r_{j}}{\psi_{j}r_{max}^{2}};\;\frac{\partial \psi_{j}}{\partial z}=\frac{z-z_{j}}{\psi_{j}z_{max}^{2}},$$

(19) $$\frac{\partial^{2}\psi_{j}}{\partial r^{2}}=\frac{1}{\psi_{j}r_{max}^{2}}-\frac{{\left(r-r_{j}\right)}^{2}}{\psi_{j}^{3}r_{max}^{4}};\;\frac{\partial^{2}\psi_{j}}{\partial z^{2}}=\frac{1}{\psi_{j}z_{max}^{2}}-\frac{{\left(z-z_{j}\right)}^{2}}{\psi_{j}^{3}z_{max}^{4}},$$

(20) $$\frac{\partial^{2}\psi_{j}}{\partial r\partial z}=-\frac{\left(r-r_{j}\right)\left(z-z_{j}\right)}{\psi_{j}^{3}r_{max}^{2}z_{max}^{2}}.$$

The monomials are defined as $\vartheta_{1}(x)=1,\vartheta_{2}(x)=r-r_{j},\vartheta_{3}(x)=z-z_{j}$ and have the following trivial derivatives

(21) $$\frac{\partial \vartheta_{1}}{\partial r}=0;\;\frac{\partial \vartheta_{2}}{\partial r}=1;\;\frac{\partial \vartheta_{3}}{\partial r}=0;\;\frac{\partial \vartheta_{1}}{\partial z}=0;\;\frac{\partial \vartheta_{2}}{\partial z}=0;\;\frac{\partial \vartheta_{3}}{\partial z}=1,$$

(22) $$\frac{\partial^{2}\vartheta_{1}}{\partial r^{2}}=0;\;\frac{\partial^{2}\vartheta_{2}}{\partial r^{2}}=0;\;\frac{\partial^{2}\vartheta_{3}}{\partial r^{2}}=0;\;\frac{\partial^{2}\vartheta_{1}}{\partial z^{2}}=0;\;\frac{\partial^{2}\vartheta_{2}}{\partial z^{2}}=0;\;\frac{\partial^{2}\vartheta_{3}}{\partial z^{2}}=0,$$

(23) $$\frac{\partial^{2}\vartheta_{1}}{\partial z\partial r}=0;\;\frac{\partial^{2}\vartheta_{2}}{\partial z\partial r}=0;\;\frac{\partial^{2}\vartheta_{3}}{\partial z\partial r}=0.$$

The numerical issue of division by zero at r=0 is handled by introducing a small non-zero parameter, $\varepsilon_{r=0}$, in the denominator. Specifically, the $\varepsilon_{r=0}=10^{-6}$ is used, and the terms involving 1/r are evaluated using $1/(r+\varepsilon_{r=0})$. This prevents numerical singularities while keeping the value of $\varepsilon_{r=0}$ sufficiently small does not affect the physical solution.

## 4. Numerical Example

### 4.1. Geometry

**Figure 1:**
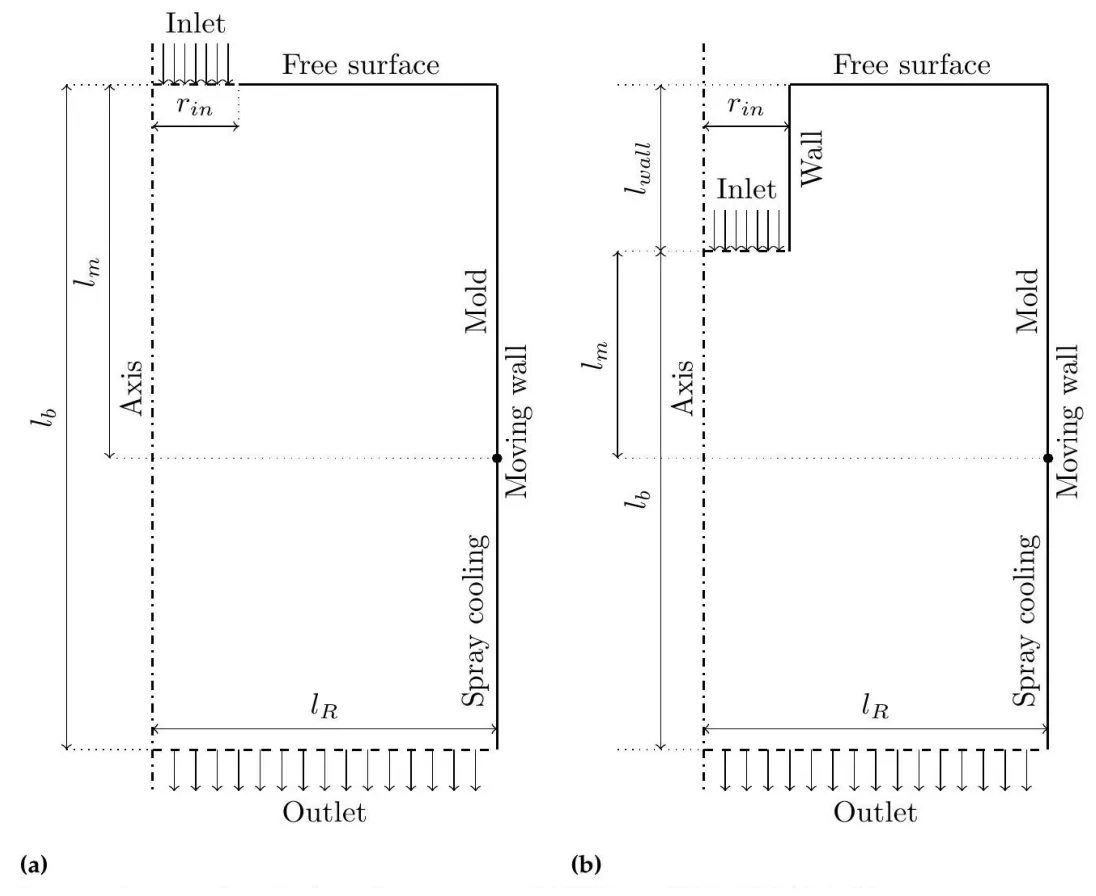
Scheme of a vertical continuous caster. (**a**) Without considering SEN; (**b**) With SEN. The dotted line (-.-.-.) represents the symmetry axis.

In a numerical example, continuous casting with vertical cylindrical geometry is observed. Because the geometry of the caster is symmetric about the vertical axis and the equations are time-averaged, an axisymmetric computational domain was constructed, and the example is solved using axisymmetric operators [32]. The scheme of vertical caster geometry with and without a submerged entry nozzle (SEN) is shown in Figure 1.

The computational domain of the round billet is 1.8 m long and 0.07 m wide, which coincides with the billet radius $l_{R}$. The molten metal is poured into the mold through the circular inlet with a radius of 0.0175 m. The upper part of the computational domain represents the molten and solidifying metal inside the mold, and the lower part represents the area with secondary spray cooling, where the outer solidified metal shell is cooled with room-temperature water and is strong enough to support the steel–liquid core. In the second scheme (Figure 1), the entry nozzle (SEN) is submerged for $l_{\mathrm{wall}\;}$. The computational domain dimensions for the billet are listed in Table 1.

### 4.2. Boundary Conditions

At the top, an inlet boundary condition is prescribed, with the liquid metal poured into the mold, and a free-surface boundary condition is applied at the meniscus. The Dirichlet boundary condition is prescribed for velocity, temperature, turbulent kinetic energy, and dissipation rate at the inlet. The molten metal is poured from the cylindrical SEN and is assumed to have only a vertical component $u_{z,\;\mathrm{in}\;}=u_{\mathrm{cast}\;}r_{\mathrm{out}\;}^{2}/r_{\mathrm{in}\;}^{2}$ and $u_{r,in}=0\;m/s$. The inlet turbulent kinetic energy is calculated as $k_{in}=3/2{\left(Iu_{z,in}\right)}^{2}$, where $I=0.16Re^{-1/8}$ is the turbulent intensity with Re standing for Reynolds number. The value for the dissipation rate is calculated as $\varepsilon_{\mathrm{in}\;}=C_{\mu }^{3/4}k_{\mathrm{in}\;}^{3/2}/\left(0.07L_{c}\right)$, where $L_{c}$ stands for characteristic length. The temperature at the inlet is defined by the casting temperature $T_{\mathrm{in}\;}=T_{\mathrm{cast}\;}$. Neumann boundary condition ∂p/∂z=0 Pa/m is prescribed for pressure at the inlet. The free surface boundary condition at the meniscus uses Neumann boundary conditions for velocity in the radial direction $\partial u_{r}/\partial z=0{\;s}^{-1}$, temperature ∂T/∂z=0 K/m, turbulent kinetic energy $\partial k/\partial z=0\;m/s^{2}$, dissipation rate $\partial \varepsilon /\partial z=0\;m/s^{3}$, and pressure ∂p/∂z=0 Pa/m and the Dirichlet boundary condition for velocity in the vertical direction $u_{z}=0$ m/s.

The rotation axis is positioned on the left side in all plots. It uses symmetry boundary conditions, meaning Neumann boundary conditions are applied, with the radial derivative of the axial velocity set to zero $\partial u_{z}/\partial r=0{\;s}^{-1}$, temperature ∂T/∂r=0 K/m, turbulent kinetic energy $\partial k/\partial r=0\;m/s^{2}$, dissipation rate $\partial \varepsilon /\partial r=0\;m/s^{3}$, and pressure ∂p/∂r=0 Pa/m. There is no flow over the symmetry axis, so the radial velocity is set to zero $u_{r}=0\;m/s$.

At the bottom, the outlet boundary is set, and Neumann boundary conditions are prescribed for velocity $\partial u_{\zeta }/\partial z=0{\;s}^{-1}$, temperature ∂T/∂z=0 K/m, turbulent kinetic energy $\partial k/\partial z=0\;m/s^{2}$, and dissipation rate $\partial \varepsilon /\partial z=0\;m/s^{3}$. The Dirichlet boundary condition is prescribed for pressure and is set to the ambient pressure $p=p_{0}$.

The moving walls at the right side of the domain move along the casting (vertical) direction at the casting speed. The boundary condition for the velocity at the walls is set to $u_{z}=u_{\mathrm{cast}\;}$ and $u_{r}=0\;m/s$. The Neumann boundary condition is used for turbulent kinetic energy $\partial k/\partial r=0\;m/s^{2}$, dissipation rate $\partial \varepsilon /\partial r=0m/s^{3}$, and pressure ∂p/∂r=0 Pa/m. The temperature at the boundary is calculated from the heat flux $q=h_{\mathrm{HTC}\;}(z)\left(T-T_{0}\right)$ where

(24) $$h_{HTC}=\{\begin{array}{c} h_{HTC,m}=;0\leq z\leq l_{m}\\ h_{HTC,sc}=;l_{m}\leq z\leq l_{b}, \end{array}$$

stands for the heat transfer coefficient (HTC), which has different values in the mold $h_{HTC,m}$ and in the secondary cooling zone $h_{HTC,sc}$, and $T_{0}$ is the reference ambient temperature.

The no-slip boundary condition is applied for velocity at the SEN walls, and the velocity is set to $u_{\zeta }=0\;m/s$. The Neumann boundary condition is applied for pressure. The Dirichlet boundary condition is applied for temperature $T=T_{\mathrm{cast}\;}$, turbulent kinetic energy k=0, and dissipation rate ε=0.

### 4.3. Material Properties and Casting Process Parameters

In general, the material properties of steel are temperature dependent. However, in this example, the constant values in Table 2 are used to simplify the recalculation of our solution with other numerical methods. These material properties have been previously used in [2].

The values for process parameters are listed in Table 3.

## 5. Results

**Figure 2:**
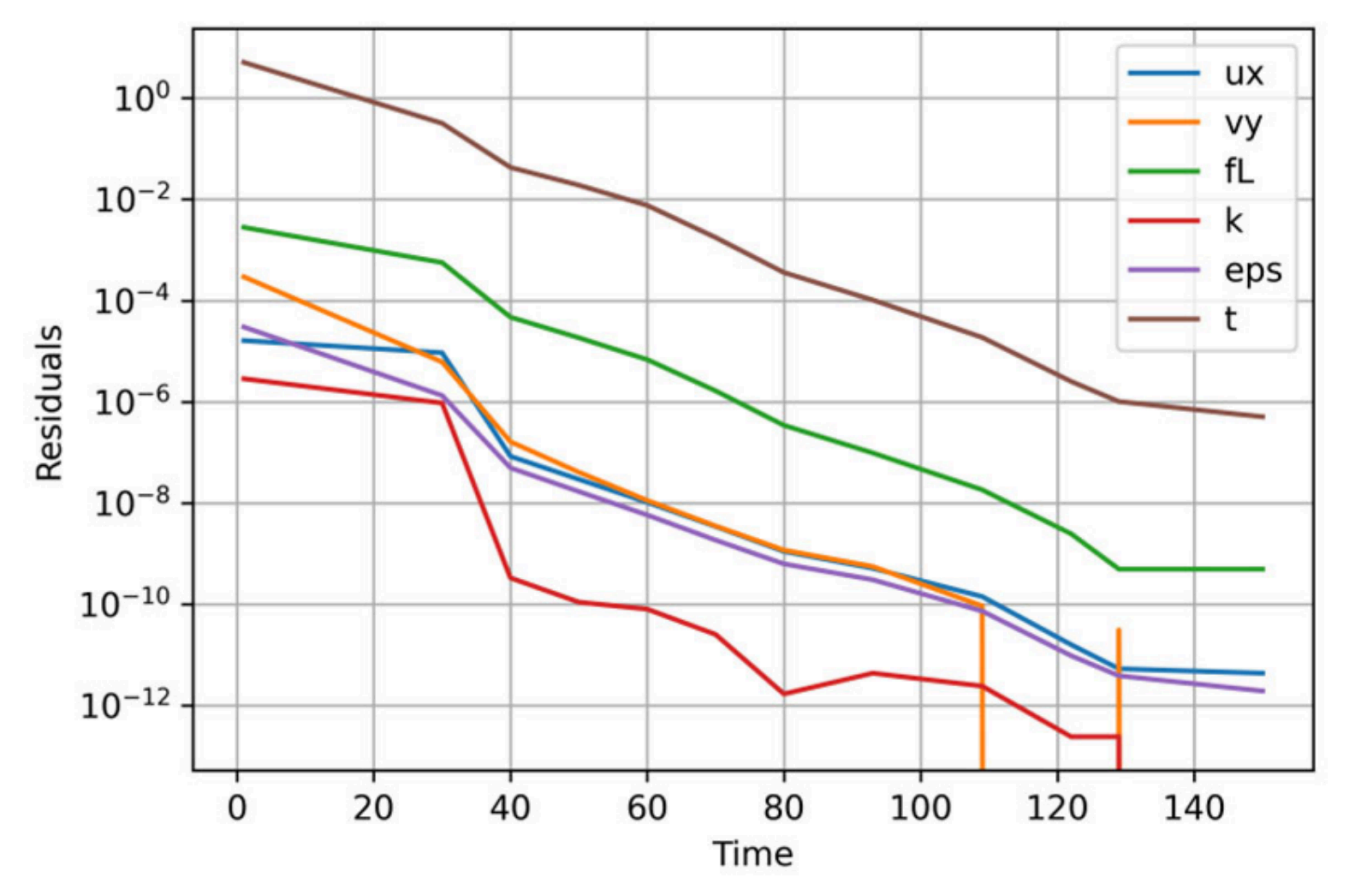
Convergence history for the representative case.

The calculation is stopped once a steady-state condition is achieved, which happens when the normalized residuals for all variables fall below $10^{-6}.$ The convergence history for a representative case (XXXL described in detail in the following sections) is shown in Figure 2.

### 5.1. Node Arrangement

Several discretization arrangements were tested to assess the dependence of the results on the node arrangement. The node arrangements involved are listed in Table 4 and range from 3563 to 123,388 nodes. The nodes are more densely clustered in areas with rapid flow changes to prevent convergence problems caused by a sparse node arrangement. The time step for the node arrangement convergence test was $\Delta t=10^{-5}\;s$. The wall-clock time of calculations is set to the approximate time between the 1st and 100th outputs and is listed in Table 4, along with calculated examples for each node arrangement. The output was written every second of the simulation.

For the LRBFCM implementation, the code was parallelized using OpenMP shared memory. The computational workload for the nodes and their corresponding local support domains was distributed across multiple CPU threads. The implementation does not employ MPI; therefore, all calculations were performed within a shared-memory environment on a single computational node.

The calculations were performed using 8 CPU threads on a system equipped with an Intel^®^ Core™ i9-9900K CPU operating at 3.60 GHz, with a maximum clock frequency of 5.00 GHz. The system uses a 64-bit operating system with an x64-based processor. The reported wall-clock times are provided as additional information on the computational effort required for the selected cases and are not intended as a direct benchmark or performance comparison with FVM software. The LRBFCM implementation currently relies solely on OpenMP parallelization, whereas the FVM implementation employs a fully parallelized approach. Consequently, the wall-clock times of the two methods are not directly comparable due to differences in their parallelization strategies and implementation. Nevertheless, the reported computational times can be used to assess the time efficiency of the LRBFCM by comparing different LRBFCM cases under consistent computational conditions. This indicates the relative computational cost and scalability of the LRBFCM approach across the investigated cases.

In node arrangements S, M, L, XL, XXL, and XXXL, the same approach as used to construct node arrangements in two-dimensional Cartesian coordinates [12] is applied. The node arrangement in the horizontal direction is divided into two areas: the inlet part, where an equidistant node arrangement is used, and the meniscus part, where nodes are condensed towards the outer wall. In the vertical direction, the nodes are concentrated at the top and the end of the mold. An alternative node arrangement, XL_GRAD, was tested for node arrangement XL. In this case, the node arrangement in the horizontal area is treated as a single zone, with nodes condensed toward the outer wall. In the vertical direction, the nodes are again condensed at the top and the end of the mold.

Additionally, a geometry including the SEN was tested (XL_SEN). In this example, additional nodes were added above the inlet plane (z=0 m). The distribution of nodes in the horizontal direction follows the approach used in XL_GRAD.

**Figure 3:**
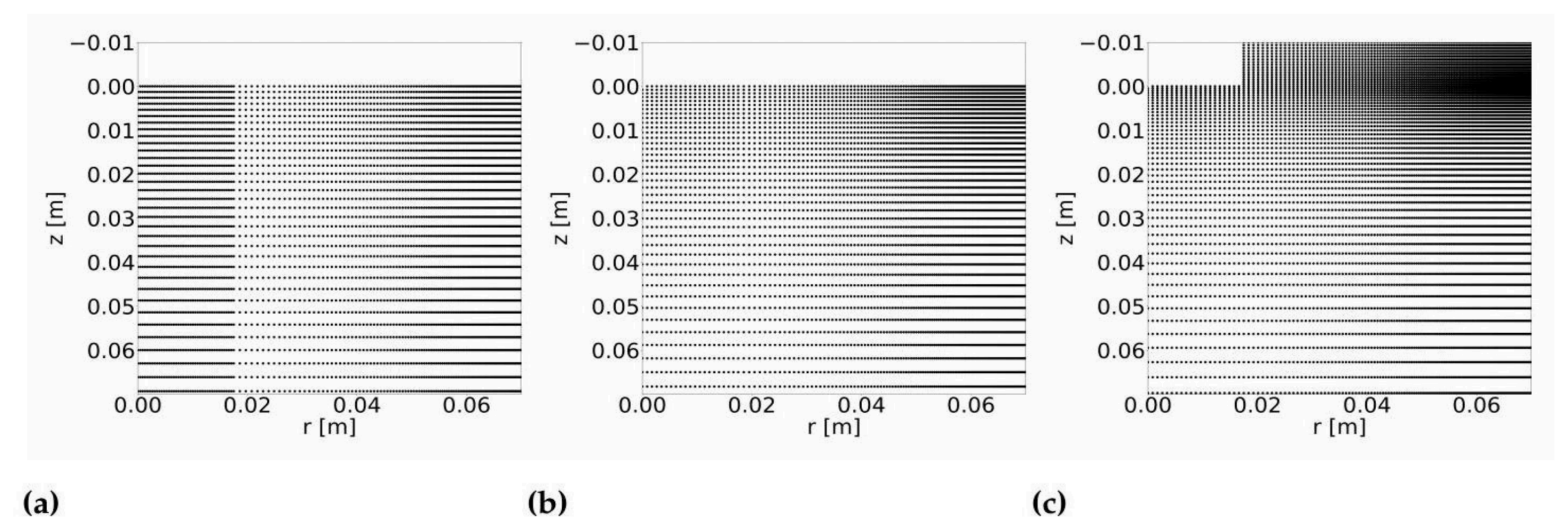
Close-up of node arrangements. (**a**) XL; (**b**) XL_GRAD; (**c**) XL_SEN.

The graphical representation of typical node arrangement segments is shown in Figure 3. The ratio between the radial and axial dimensions of the billet is $l_{R}:l_{b}=0.07$: 1.8=0.039, meaning that considerably more nodes are required in the axial direction. The node arrangements are constructed in such a way that the ratio between nodes in the radial $N_{r}$ and axial $N_{z}$ direction (without the nodes above the inlet plane for node arrangement XL_SEN) is approximately $N_{r}/N_{z}=0.47$ for all cases. The ratio between node distances in radial and axial directions is considerably smaller (see Table 5 for the ratio between minimal distances between nodes) than the desired values between 0.5 and 2. Nonetheless, the convergence of the results is ensured because nodes are farther apart in areas with s.

### 5.2. Spatial Convergence Test

The convergence of the LRBFCM simulations was systematically assessed using grid convergence criteria. The grid convergence index (GCI), in conjunction with Richardson extrapolation, was employed to quantify the discretization error and to evaluate the sensitivity of the numerical solution to the spatial resolution. It should be noted that the computational grids were refined non-uniformly to provide higher resolution in regions with strong physical gradients, such as the solidification front and flow-development regions, while avoiding unnecessary computational cost in regions with smoother solutions. Despite this non-uniform refinement, GCI analysis following [33] was applied to quantify the solution’s sensitivity to overall spatial resolution. A characteristic grid spacing

(25) $$h_{c}=\sqrt{\frac{1}{N_{\mathrm{All}\;}}A}$$

where $N_{\mathrm{All}\;}$ is the number of all nodes in the computational domain and A is the computational domain’s surface area, which was defined for each discretization level, and the corresponding grid refinement ratio $r_{\mathrm{ref}\;,ij}=h_{c,i}/h_{c,j}$ between two consecutive grids i and j, where i is the coarsest grid, determined from these characteristic resolutions. Richardson extrapolation and the corresponding GCI values were subsequently used to assess whether further spatial refinement produced only negligible changes in the selected representative quantities.

**Figure 4:**
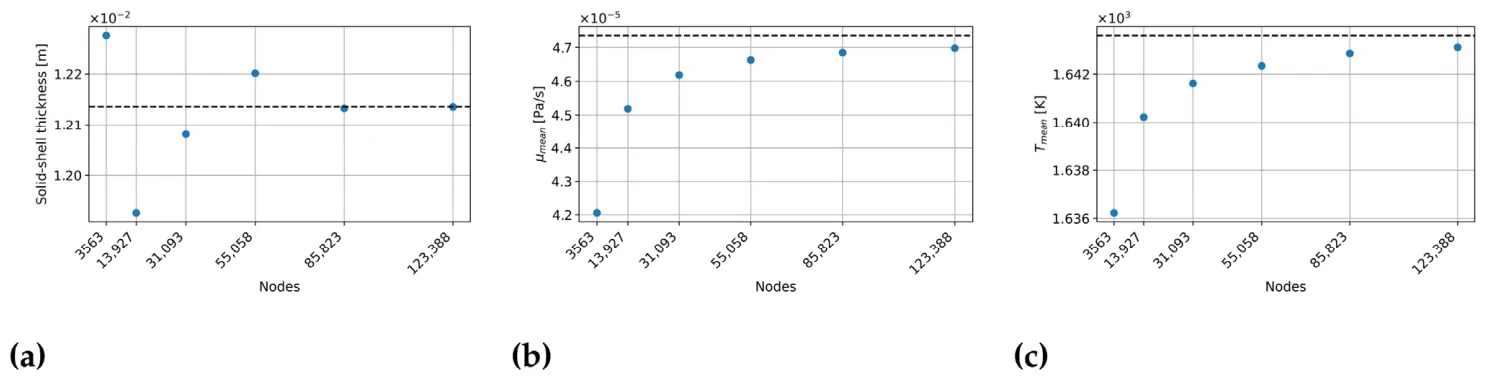
Spatial convergence test. (**a**) Solid-shell thickness at z=0.8 m; (**b**) Domain-averaged RMS value of $\mu_{t}$; (**c**) Domain-averaged RMS value of T. Richardson’s extrapolation of the observed variables is represented by a dashed line.

Because the coupled model involves solidification, turbulent fluid flow, and heat transfer, different representative quantities were selected for the GCI analysis to assess the convergence of the corresponding physical fields. For the solidification process, the solid shell thickness at the mold exit (Figure 4a) was selected as the representative quantity. This parameter was selected because the shell thickness at the mold exit directly characterizes the extent of solidification and is a key indicator of the accuracy of predicted solidification behavior in continuous casting.

The root-mean-square (RMS) value of $\mu_{t}$ (Figure 4b) over the entire computational domain was selected as the representative parameter for assessing the grid convergence of the turbulent flow field since it characterizes the additional momentum and heat transport associated with turbulent fluctuations and is therefore directly related to the characteristics of the modeled turbulent flow. The use of the RMS value provides a global measure of the predicted turbulent viscosity field and accounts for its spatial variations throughout the computational domain, rather than relying on a local value that may be strongly influenced by the flow conditions at a specific location. Consequently, the RMS turbulent viscosity serves as a representative scalar quantity for evaluating the sensitivity of the turbulent flow solution to changes in spatial resolution. It provides an overall measure of the turbulence model’s grid dependence.

For the energy equation, the domain-averaged RMS value of T (Figure 4c) was used as the representative quantity because temperature is the primary variable governing heat transfer and phase-change evolution, while a domain-wide measure reduces the influence of localized numerical variations and provides an overall assessment of thermal-field convergence. The use of separate representative quantities enables independent evaluation of the grid-convergence behavior of the principal coupled physical processes, rather than relying on a single variable.

The GCI and Richardson extrapolation values for representative quantities are presented in Table 6.

The time step used in all node convergence test examples was $\Delta t=10^{-5}\;s$. The wall-clock times for each node arrangement are listed in Table 4.

**Figure 5:**
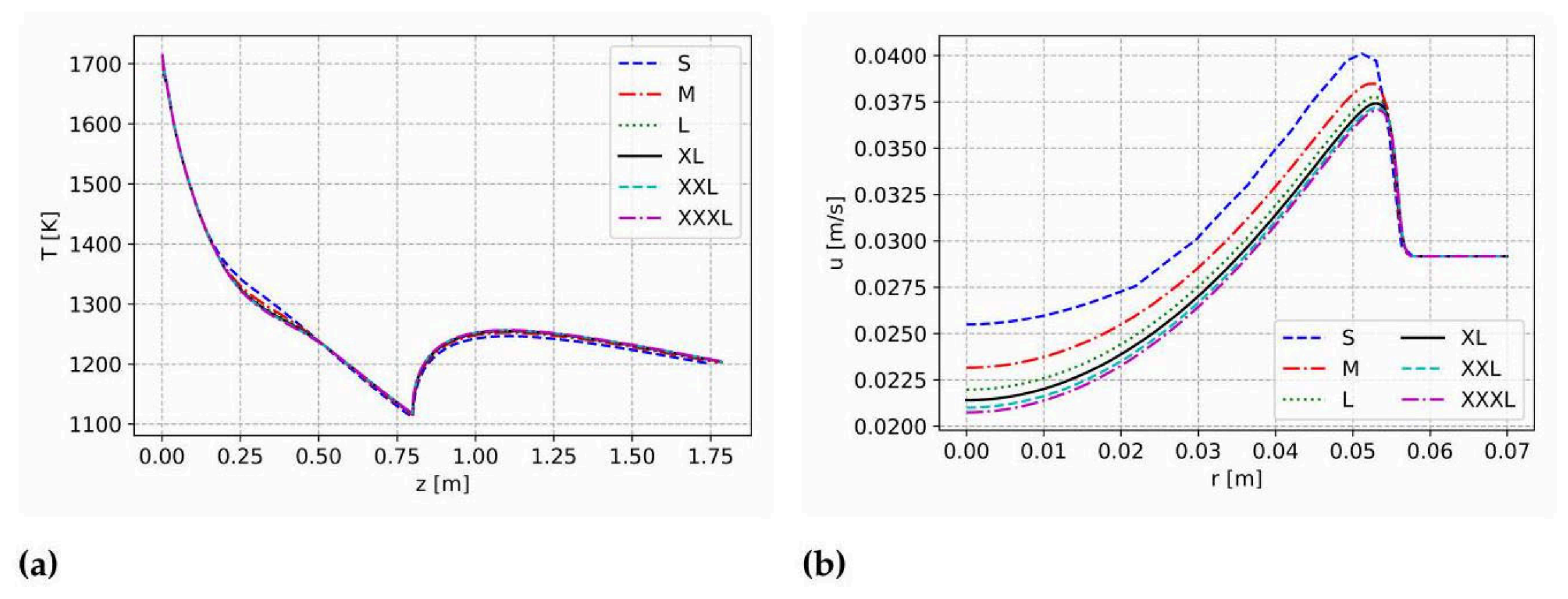
Node arrangement comparison. (**a**) Node arrangement comparison for the temperature cross-section at r=0.07 m; (**b**) Node arrangement comparison for velocity at the cross-section at z=0.8 m.

**Figure 6:**
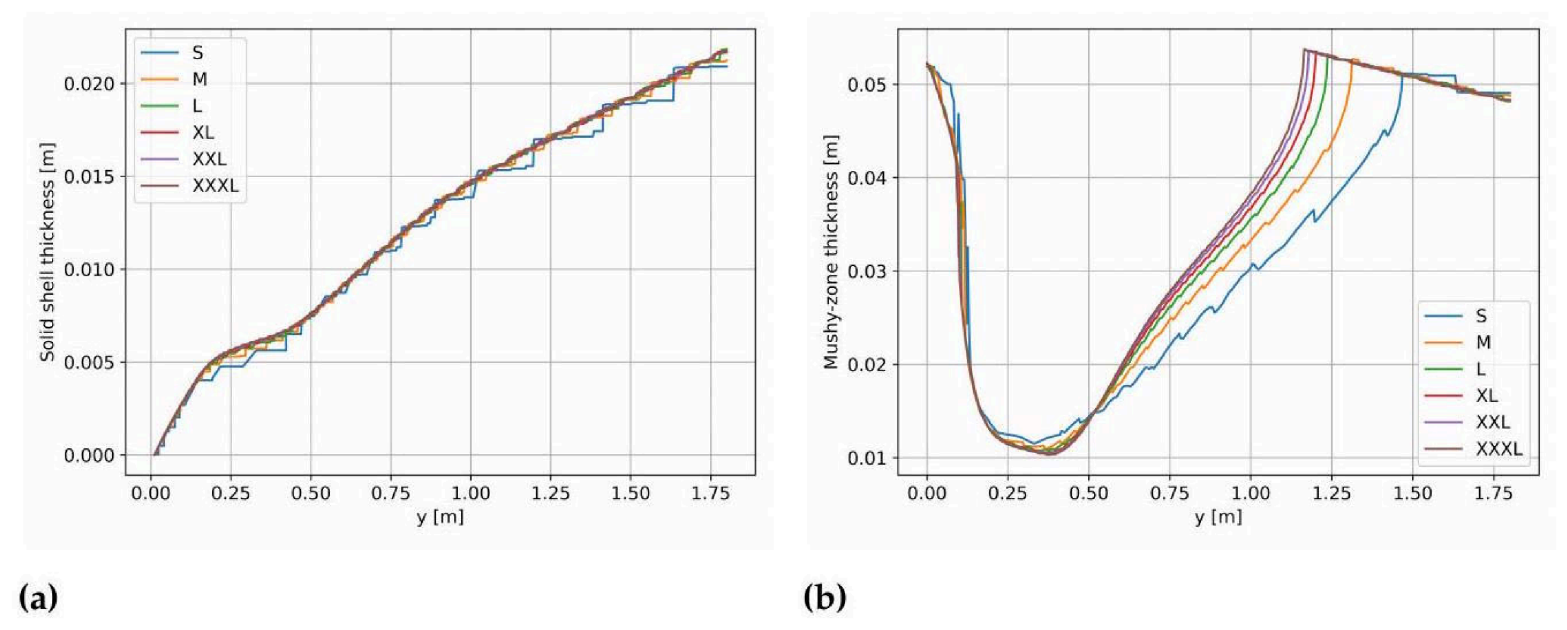
Node arrangement comparison for solidification parameters. (**a**) Node arrangement comparison for solid-shell thickness; (**b**) Node arrangement comparison for mush-zone thickness.

The velocity and temperature cross-sections for different node arrangements are shown in Figure 5, and the evolution of the solid-shell and mushy-zone thickness during the solidification process is presented in Figure 6. The solid-shell thickness is defined as the distance between the wall and the location where the $f_{L}$ reaches 0.01 and represents the solidified material adjacent to the wall. In contrast, the mushy-zone thickness corresponds to the region where the $f_{L}$ varies between 0.01 and 0.99, indicating the extent of the phase-change region separating the solid shell from the liquid core.

**Figure 7:**
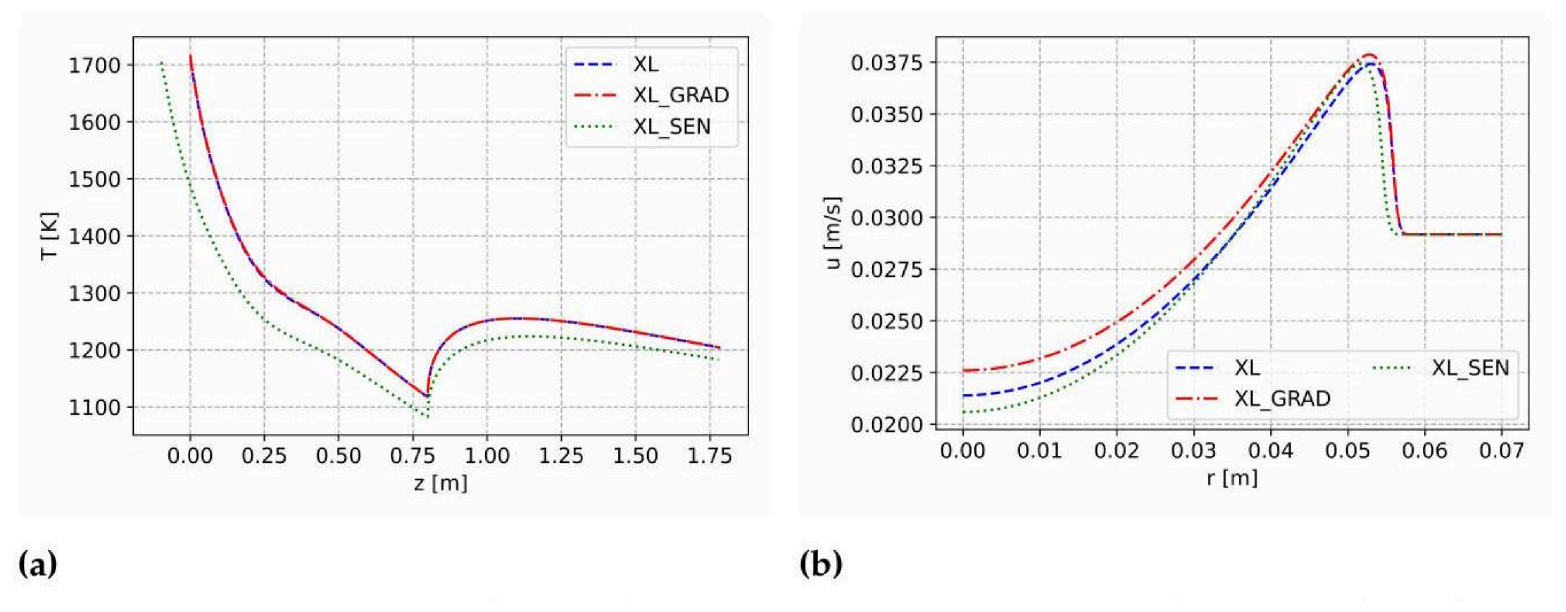
Results at node arrangements XL, XL_GRAD, and XL_SEN. (**a**) Node arrangement comparison for the temperature cross-section at r=0.07 m; (**b**) Node arrangement comparison for velocity at the cross-section at z=0.8 m.

Due to the long wall-clock times for denser node arrangements, the influence of node arrangement density and geometry was tested on intermediate node arrangement XL. The velocity and temperature cross-sections for XL, XL_GRAD, and XL_SEN node arrangements are shown in Figure 7. The time step was set to $\Delta t=10^{-5}\;s$. The wall-clock times for each node arrangement are listed in Table 4. The influence of node arrangement on temperature is negligible, as seen in Figure 7a. On the other hand, the SEN submergence depth has a considerable influence on the temperature field. The velocity cross-section at the end of the mold shows that the density distribution of the node arrangement can slightly influence the velocity field at the billet center.

**Figure 8:**
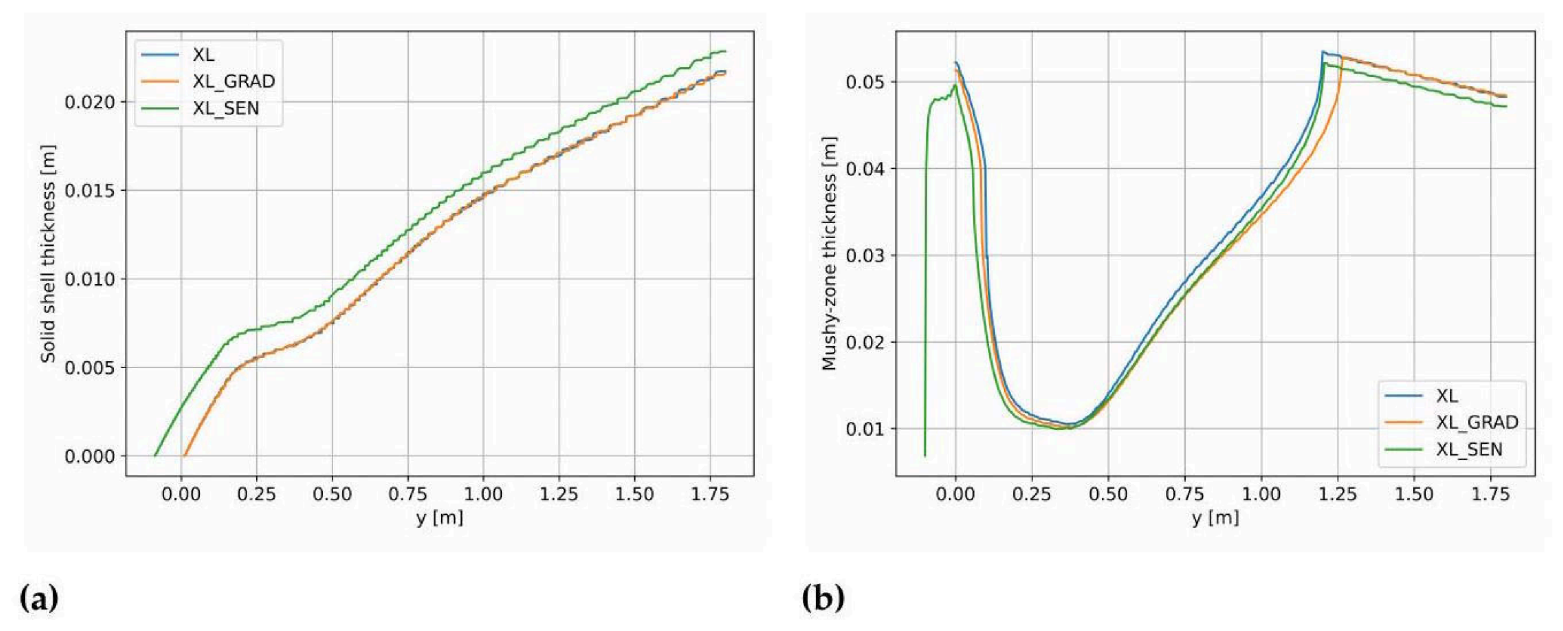
Results at node arrangements XL, XL_GRAD, and XL_SEN. (**a**) Node arrangement comparison for solid-shell thickness; (**b**) Node arrangement comparison for mushy zone thickness.

The evolution of solid-shell and mushy-zone thicknesses along the vertical direction are shown in Figure 8. In XL_SEN, the solid-shell thickness develops earlier as the inlet is below the meniscus. The XL and XL_GRAD solid-shell thicknesses are very similar over most of the domain, indicating that the node arrangement near the center has little influence on the solidification near the outer walls. Downstream of the inlet, all three cases exhibit a similar overall trend in mushy-zone thickness. The XL_SEN case predicts slightly lower values in the region below z≈0.4 m. In contrast, the XL_GRAD case shows a slight extension of the mushy zone in the axial direction, indicating a deeper penetration of the solidification front.

**Figure 9:**
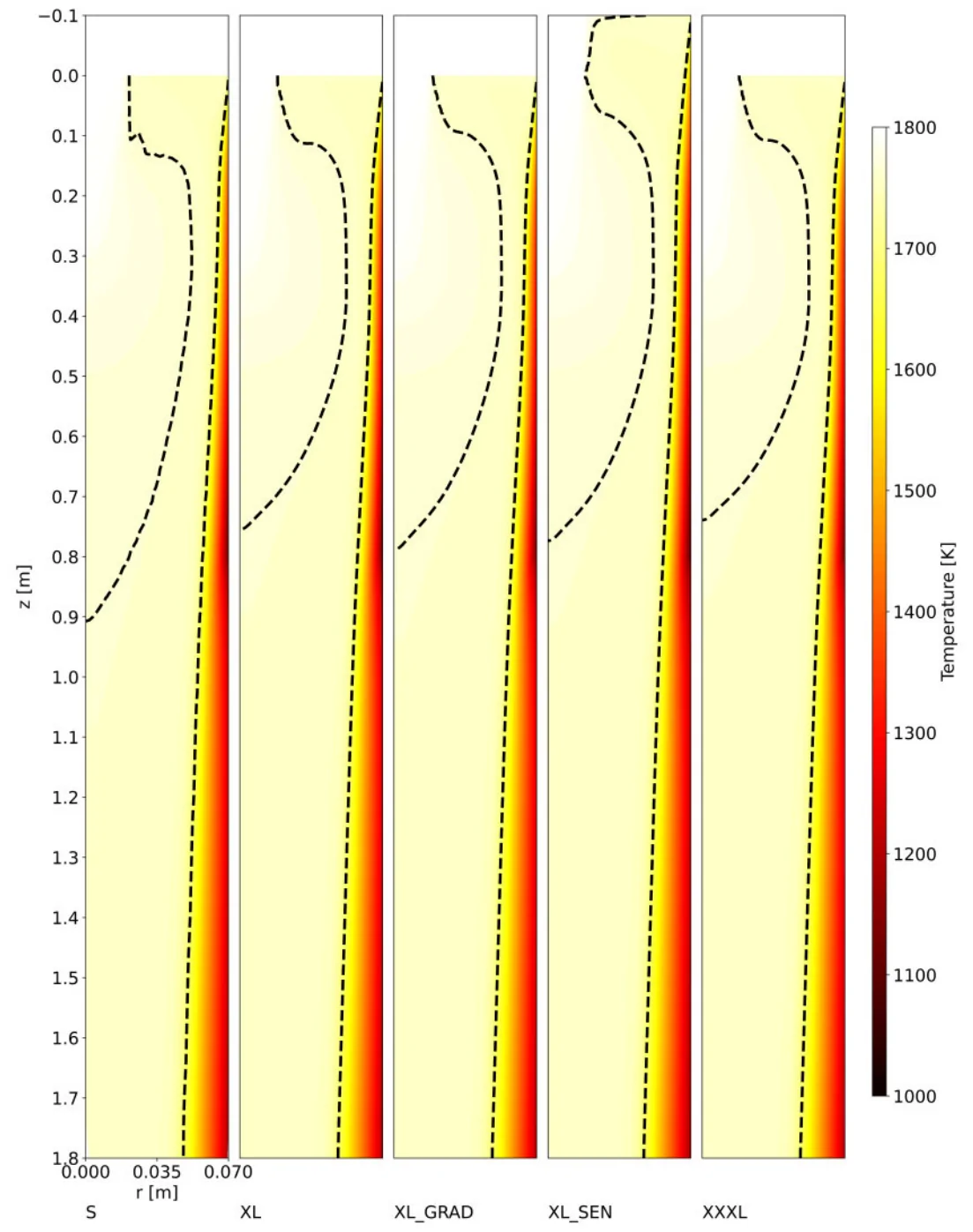
Temperature field comparison for different node arrangements. The dotted lines represent the liquidus (left) and solidus (right) lines.

**Figure 10:**
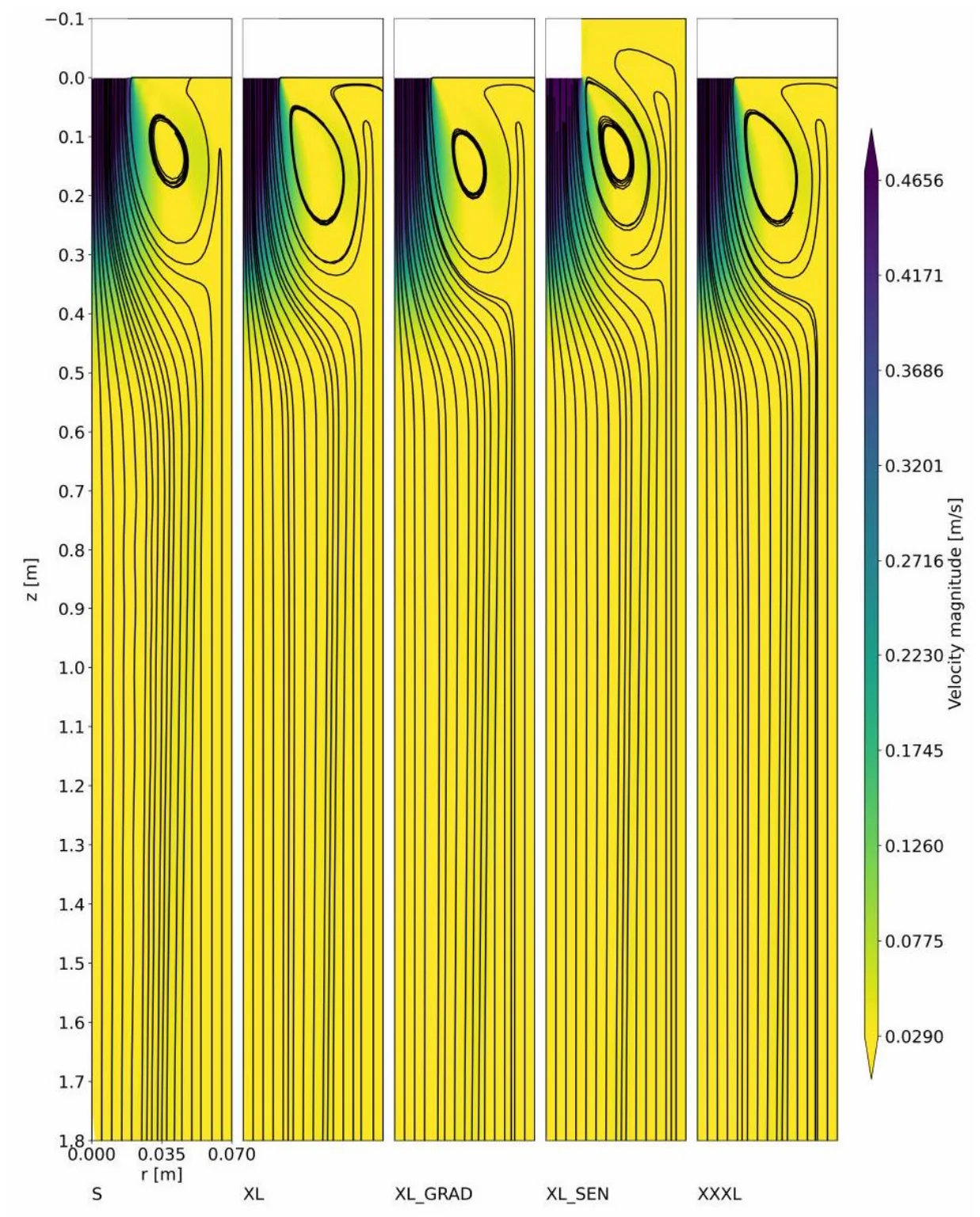
Streamlines and velocity field comparison for different node arrangements.

**Figure 11:**
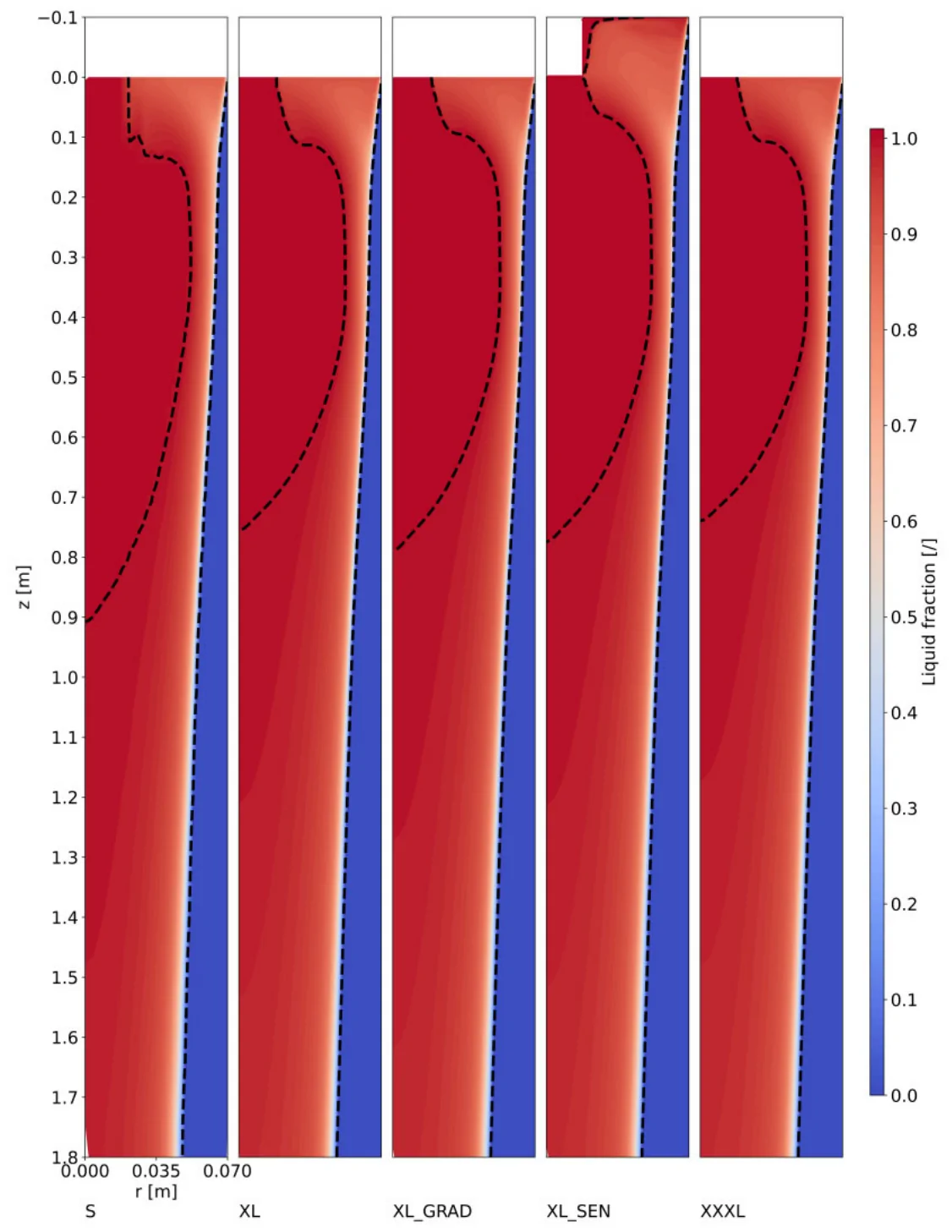
Liquid fraction field comparison for different node arrangements. The dotted lines represent the liquidus (left) and solidus (right) lines.

Figure 9 shows temperature, Figure 10 shows velocity magnitude and streamlines, and Figure 11 shows liquid fraction fields for different node arrangements. The S case shows a deeper penetration of the liquidus isotherm, whereas the XXXL case exhibits a comparatively shallower liquidus line. Among the XL, XL_GRAD, and XL_SEN cases, the temperature fields are largely consistent, with the XL_GRAD case showing a slightly lower position of the liquidus line at the center. The velocity field shows a recirculation region downstream of the inlet in all cases. The overall flow structure is qualitatively similar, with the main flow developing predominantly in the axial direction and a localized recirculation zone near the upper part of the domain. However, the extent and shape of the recirculation region vary between the different cases. The XL_SEN case shows the most pronounced recirculation, while XL and XL_GRAD produce smaller regions. Further downstream, the streamlines become predominantly parallel, and the velocity fields are highly similar among the cases, indicating that the differences are primarily localized to the inlet region. The liquid-fraction distributions exhibit the same overall behavior as the temperature isotherms. The different cases show similar downstream trends, with only minor variations in the position of the liquidus and solidus regions.

### 5.3. Time Step

The time step length was tested on the smallest node arrangement, S, with 3563 nodes, since the computational time for larger arrangements would be extremely long. Case $dt_{8}$ with a time step $\Delta t=10^{-5}\;s$ corresponds to case S in Table 4. Although this node arrangement is too sparse to yield exact results, it is accurate enough for rough approximation and appropriate for testing, as it provides a solution in a relatively short time. Wall-clock time is listed in Table 6 along with calculated examples. The output results were written every second of the simulation.

### 5.4. Temporal Convergence Test

**Figure 12:**
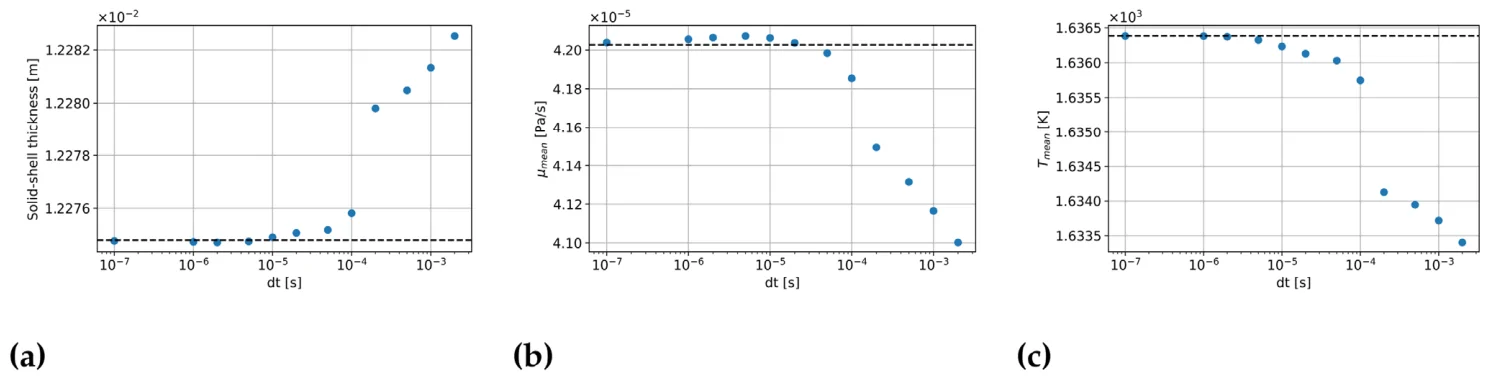
Temporal convergence test. (**a**) Solid-shell thickness at z=0.8 m; (**b**) Domain-averaged RMS value of $\mu_{t}$; (**c**) Domain-averaged RMS value of T. Richardson’s extrapolation of the observed variables is presented with a dashed line.

Similar to the GCI, temporal convergence was systematically investigated by introducing the time convergence index (TCI), in conjunction with Richardson extrapolation, to quantify the time-discretization error and evaluate the sensitivity of the numerical solution to the temporal resolution. The TCI analysis, as in [33], was applied to quantify the solution’s sensitivity to the overall temporal resolution. Similar to the spatial refinement resolution, the temporal refinement ratio $r_{\mathrm{tref}\;,ji}=$ ${\Delta t}_{c,i}$/${\Delta t}_{c,j}$ between two consecutive time step sizes i and j, where i is the larger time step, was defined. Richardson extrapolation and the corresponding TCI values were subsequently used to assess temporal convergence of the selected representative quantities (the solid-shell thickness at the mold exit, $\mu_{t_{RMS}}$, and $T_{\mathrm{RMS}\;}$). The results are presented in Figure 12.

The TCI and Richardson extrapolation values for representative quantities are presented in Table 7.

**Figure 13:**
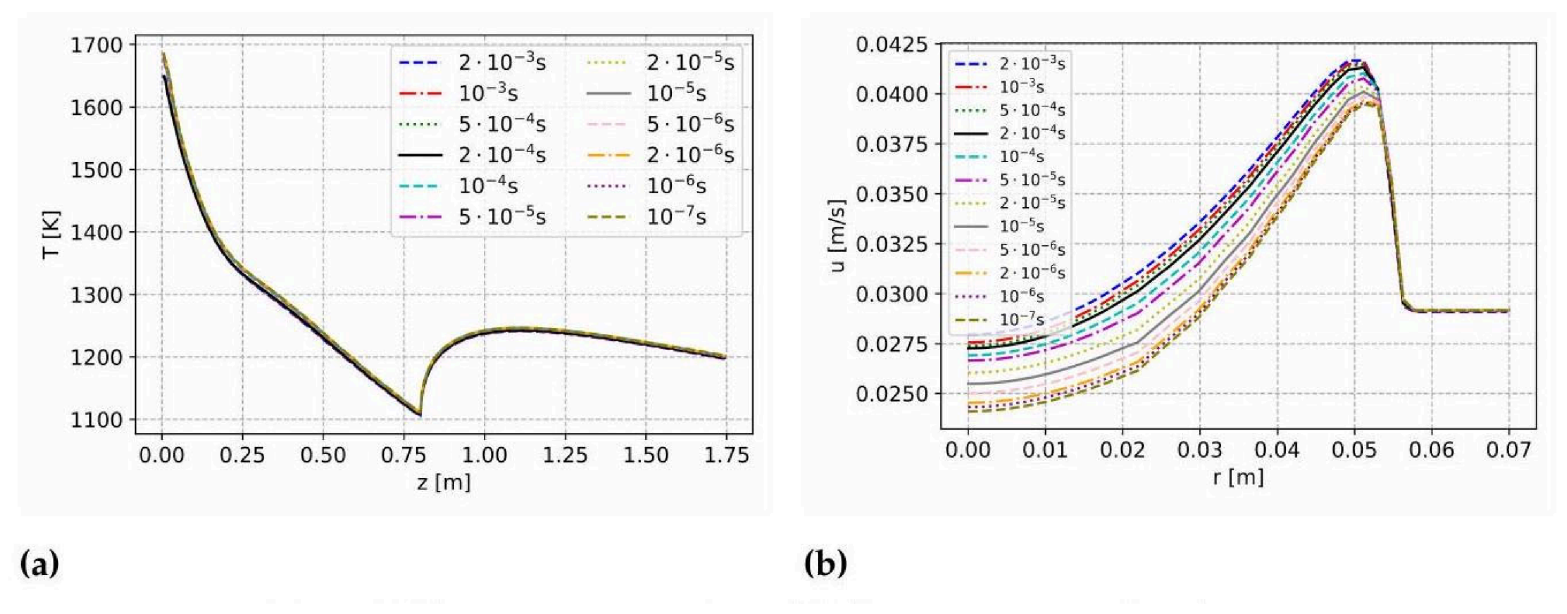
Time step comparison. (**a**) Time step comparison for the temperature cross-section at r=0.07 m; (**b**) Time step comparison for velocity at the cross-section at z=0.8 m.

**Figure 14:**
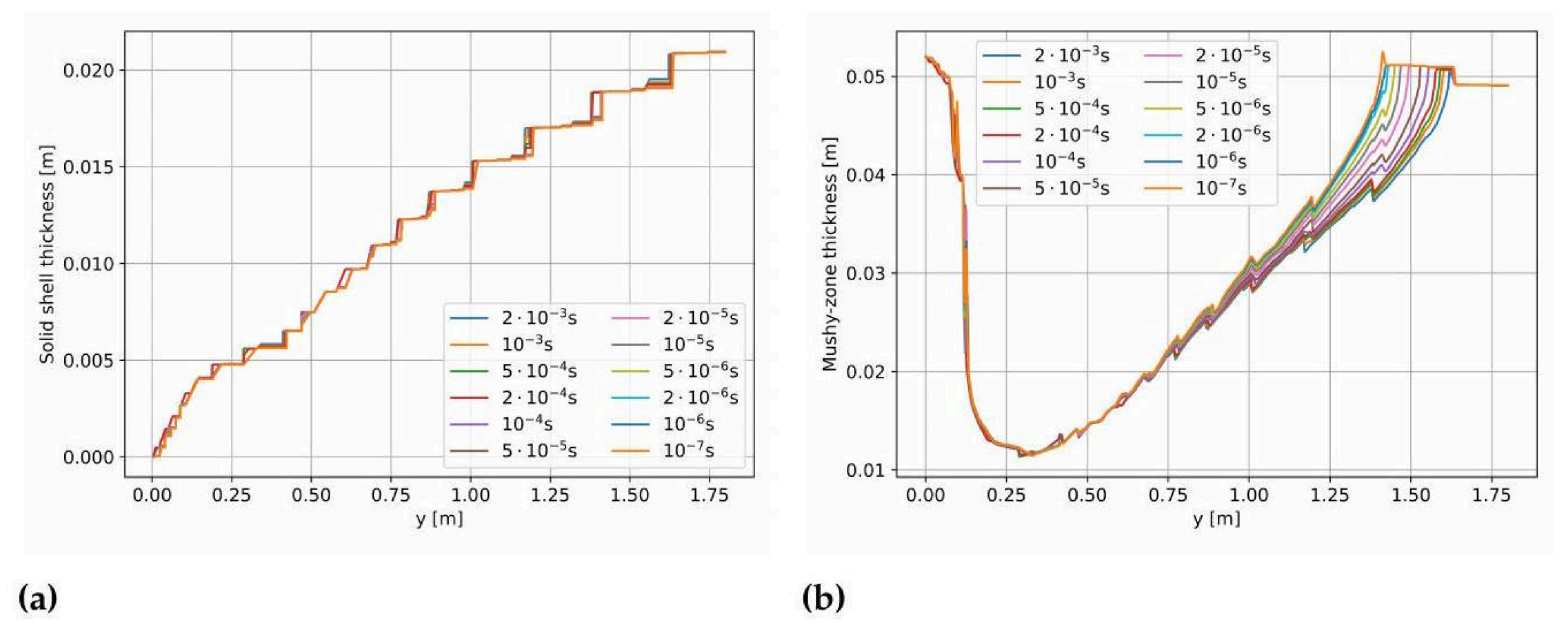
Time step comparison for solidification parameters. (**a**) Time step comparison for solid-shell thickness; (**b**) Time step comparison for mushy-zone thickness.

Figure 13 and Figure 14 show the dependence of the results on the time step. The increase in time step has little influence on the surface temperature (Figure 13a) and the solid-shell thickness (Figure 14a). Despite the use of a small time step, minor differences remain in the velocity (Figure 13b) and mushy-zone thickness (Figure 14b) at corresponding locations. However, the TCI values indicate that these differences are small relative to the overall solution and that further temporal refinement is unlikely to produce significant changes in the selected quantities. Thus, the observed discrepancies can be considered negligible for the present analysis.

### 5.5. Stability Analysis

In addition to spatial and temporal convergence criteria, the numerical stability of the LRBFCM formulation was examined using the von Neumann [34] and the Courant–Friedrichs–Lewy (CFL) [35] stability criteria. Both depend on the time step Δt size and node arrangement distances Δx;x=r,z. The minimal values for each node arrangement and the ratio between distances in radial and axial directions are listed in Table 8.

#### 5.5.1. Von Neumann Stability

The von Neumann stability criterion is defined as

(26) $$C_{vN}=\frac{\alpha_{T}\Delta t}{(\Delta x{)}^{2}}\leq 1/2$$

where $\alpha_{T}=\lambda /\left(c_{p}\rho \right)$. As the smallest Δx(x=r,z) produces the largest $C_{vN}$ and all the values must be below this value, the $C_{vN}$ is calculated only for the minimum Δx values in each direction.

**Figure 15:**
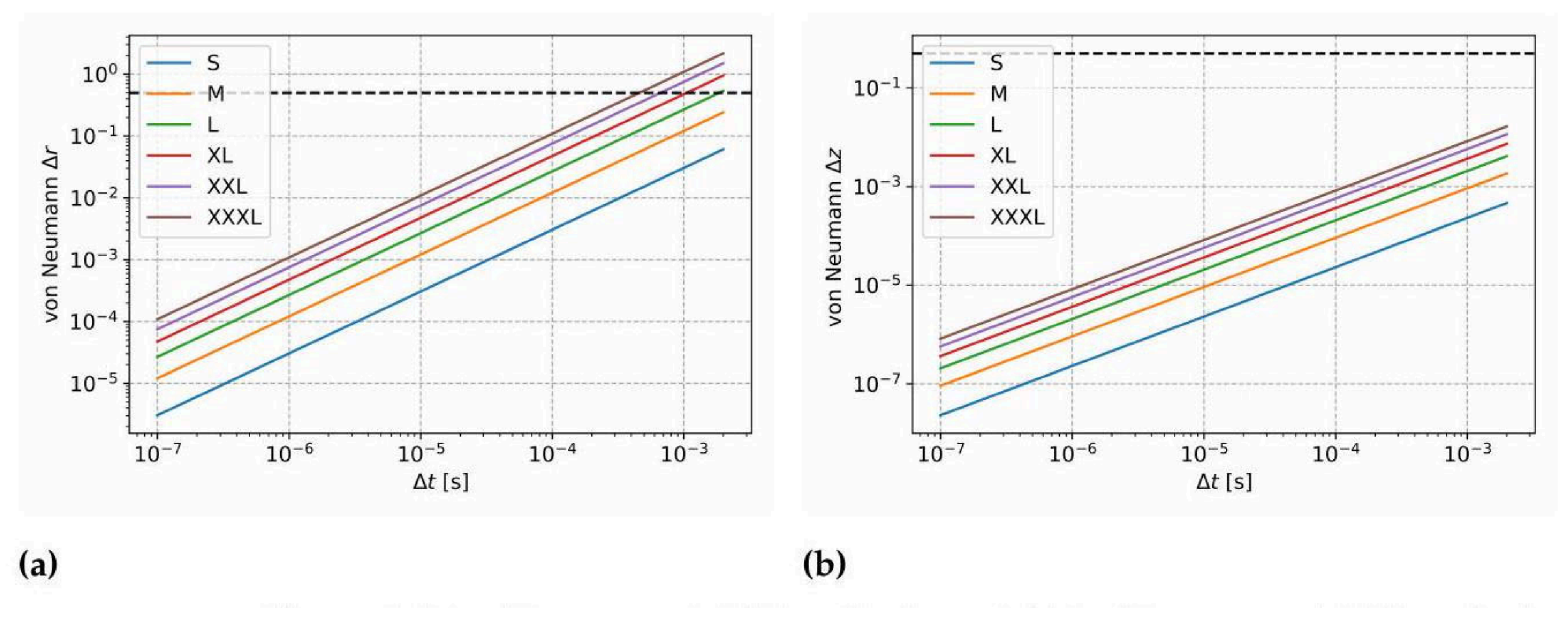
Von Neumann stability criterion. (**a**) Von Neumann stability criterion for Δr; (**b**) Von Neumann stability criterion for Δz.

This stability criterion provides a practical restriction on the time step node distance ratio to ensure stable treatment of thermal diffusion in the corresponding discretization of the energy conservation equation. The values are listed in Table 9 for the radial direction and in Table 10 for the axial direction and presented in Figure 15.

#### 5.5.2. Courant–Friedrichs–Levy Stability

**Figure 16:**
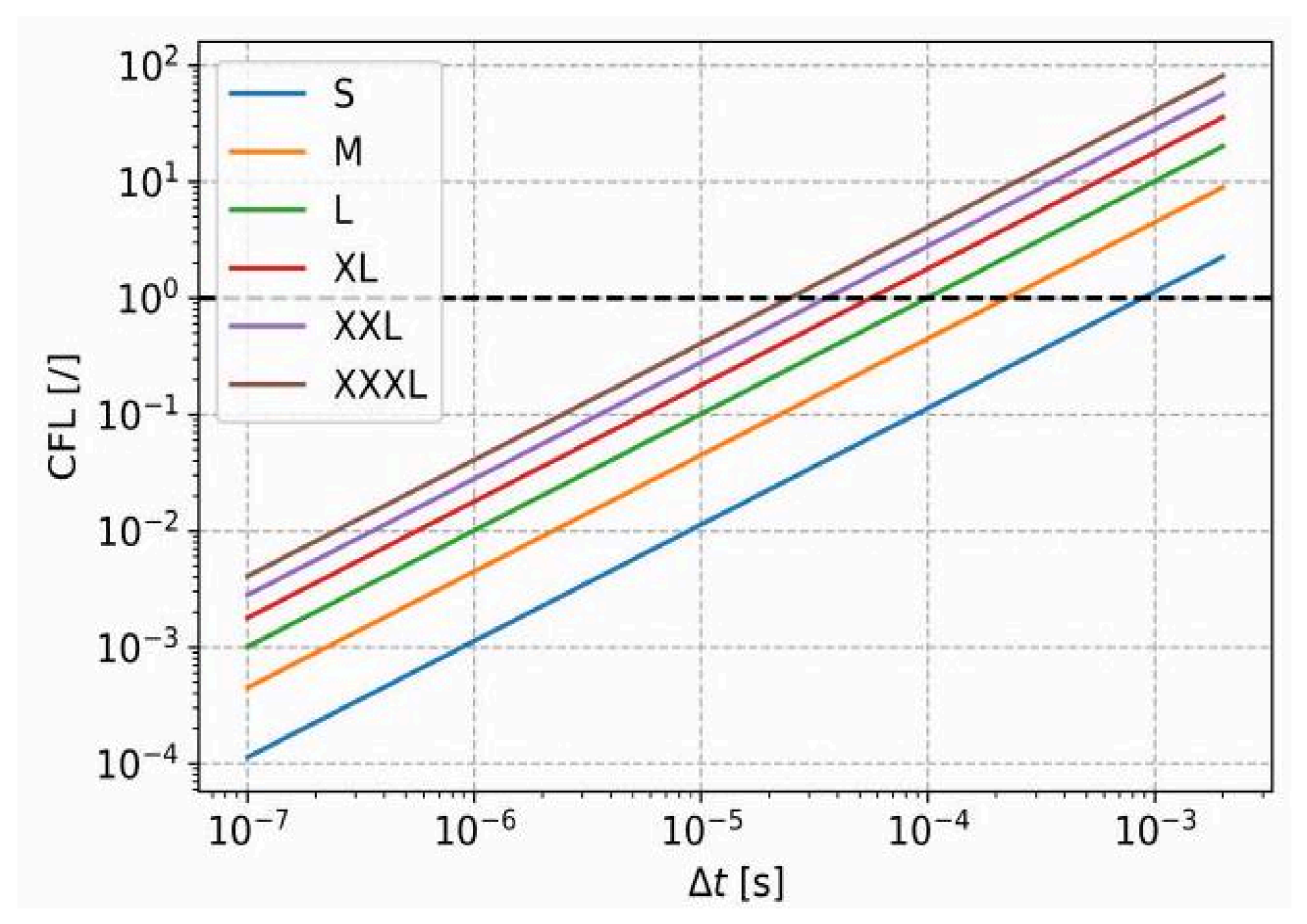
CFL stability criterion.

CFL stability criterion is defined as

(27) $$C_{CFL}=\Delta t\left(\sum_{i=1}^{n}\frac{u_{i}}{\Delta x_{i}}\right)=\frac{u_{r}\Delta t}{\Delta r}+\frac{u_{z}\Delta t}{\Delta z}\leq C_{CFLmax}=1$$

and tests the stability of the convection terms. The results for the CFL criterion are listed in Table 11 and shown in Figure 16.

The von Neumann and CFL stability criteria considered here primarily assess the stability of the individual diffusion and convection terms and do not necessarily guarantee the stability or convergence of the fully coupled governing equation. Therefore, although the tables and figures may indicate that certain individual examples are expected to converge or diverge based on these criteria, the behavior of the final coupled system may differ once all terms and their interactions are included. The combined effects of the various terms can alter the overall stability characteristics of the discretized equation; thus, convergence or divergence of the complete system should be assessed separately.

In LRBFCM, both criteria were used to impose a practical time-step node-arrangement ratio restriction for numerical discretization, rather than a complete stability requirement. Together with acceptable GCI and TCI values and convergence toward the Richardson-extrapolated solution, it is ensured that the selected spatial and temporal discretization provides numerically reliable and sufficiently grid-independent predictions of the turbulent flow, thermal field, and solidification behavior.

### 5.6. Comparison with Reference Results

The results obtained with LRBFCM were compared with those obtained with classical FVM. The differences between the two approaches were quantified by comparing velocity and temperature fields along horizontal and vertical cross-sections at characteristic locations. The numerical methodology employed here has previously been tested and validated against several established benchmark cases reported in the literature, demonstrating its ability to reproduce known physical behavior and benchmark solutions [16,17,18]. The axisymmetric problem was solved using the Fractional step method (FSM) for pressure-velocity coupling, with the k−ε turbulence model and the same parameters as those used in the meshless method. The time integration was performed using the first-order forward Euler method, and the space discretization used a second-order scheme for the convection and diffusion terms. The simulations were performed on the mesh (XXL), where cell centers coincide with points in the meshless method.

**Figure 17:**
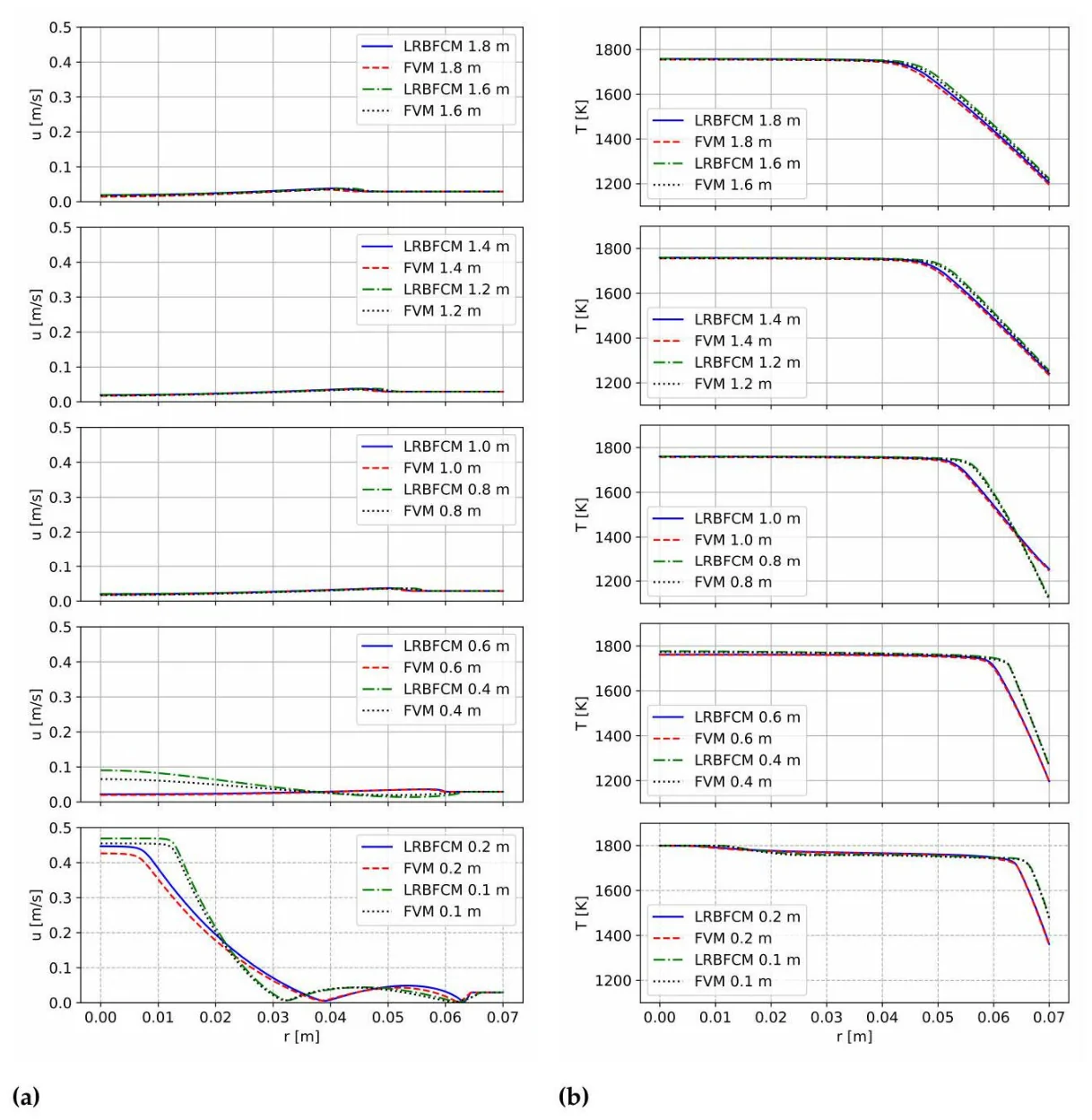
Comparison of LRBFCM and FVM results in the horizontal direction. (**a**) Velocity field comparison at different cross sections in the horizontal direction; (**b**) Temperature field comparison at different cross sections in the horizontal direction.

**Figure 18:**
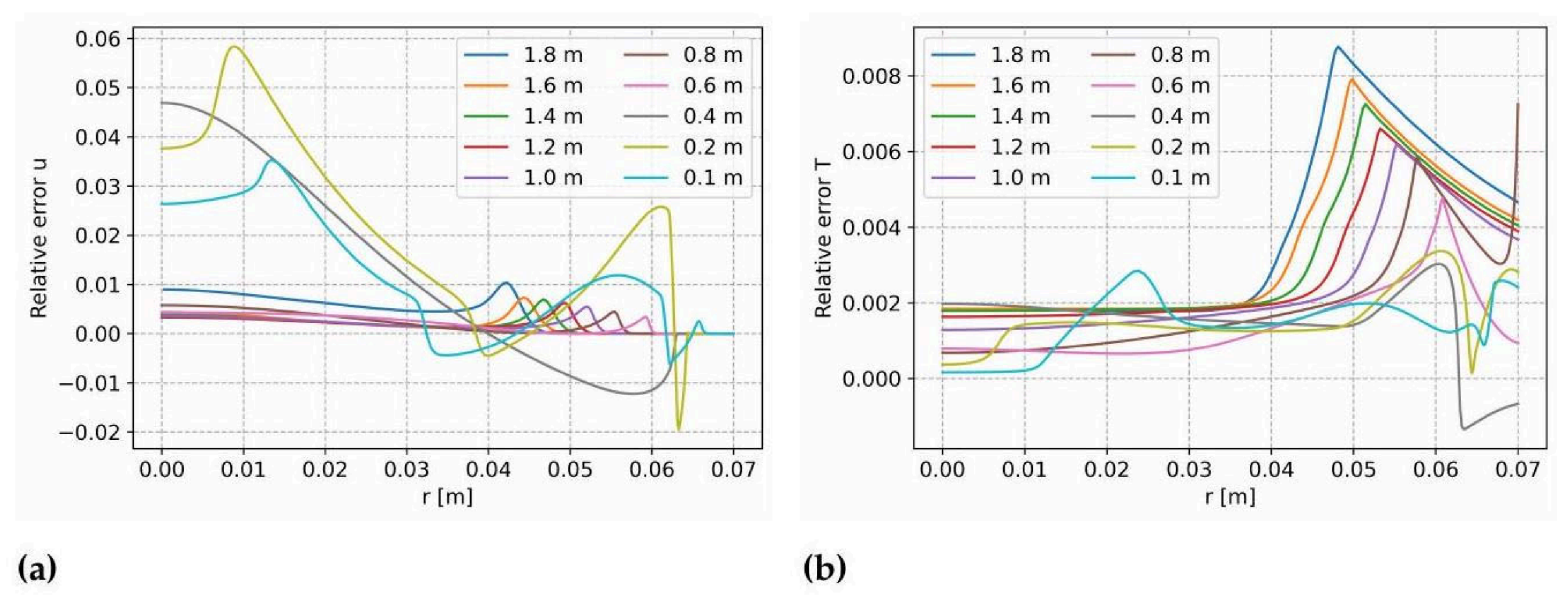
Relative error. (**a**) Relative error for velocity cross-sections in the horizontal direction; (**b**) Relative error for temperature cross-sections in the horizontal direction.

The horizontal cross-sections were compared at z=0.1 m,z=0.2 m,z=0.4 m, z=0.6 m,z=0.8 m, which corresponds to the end of the mold, z=1.0 m,z=1.2 m, z=1.4 m,z=1.6 m, and z=1.8 m, which coincides with the bottom of the computational domain. The largest discrepancies occur in the upper part of the computational domain, either within the mushy zone in the mold region. Figure 17a shows a comparison of horizontal cross-sections for velocity, and Figure 18a shows the relative error of said comparison. The FVM results predict slightly lower velocities at local extremes, which appear primarily in the mushy zone and at the center of the billet, as compared with the LRBFCM solution. In these regions, the FVM approach yields somewhat smaller peak values, while the overall flow pattern and distribution remain consistent between the two methods. Quantitatively, the maximum difference in velocity is about 0.03 m/s, occurring near the top of the domain, where the velocities are the highest, and about 0.005 m/s occurring near the bottom of the computational domain.

Figure 17b shows horizontal temperature cross-sections, with the relative error shown in Figure 18b. In this case, the FVM predicts lower temperatures than the LRBFCM approach at evaluated cross-sections. The maximum temperature difference reaches approximately 15 K, and this peak deviation is observed in the mushy zone near the top of the calculation domain, where solidification is actively progressing and thermal gradients are strongest. Outside this region, the temperature differences decrease and remain relatively small (2.5 K), indicating good agreement between the methods in fully liquid or fully solid regions. At the bottom of the computational domain, the maximum temperature difference is about 5 K.

**Figure 19:**
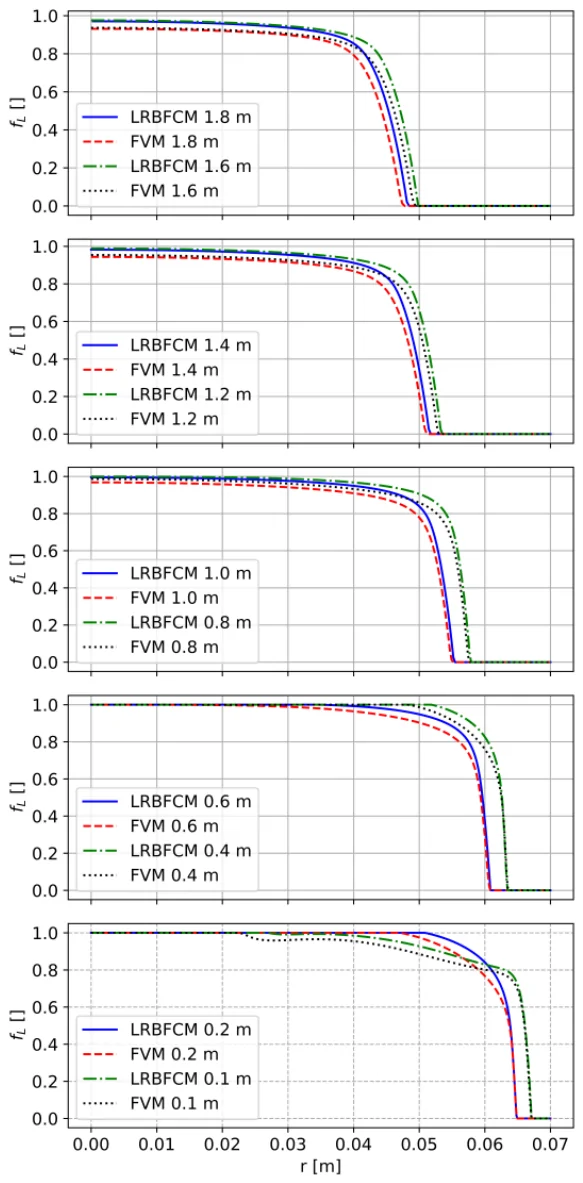
Comparison of LRBFCM and FVM results for liquid fraction field at different cross sections in horizontal direction.

**Figure 20:**
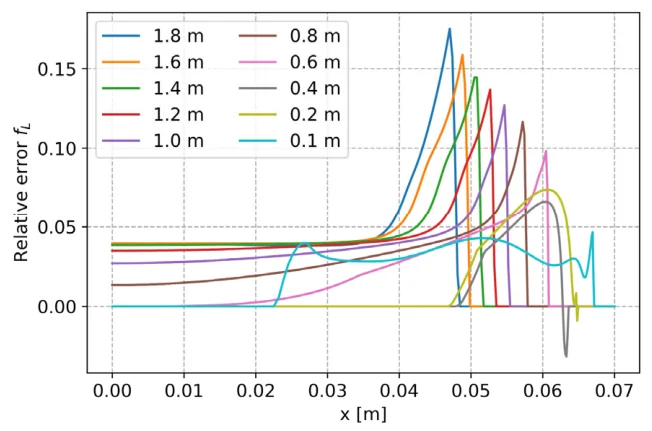
Relative error for liquid fraction cross-sections in the horizontal direction.

Figure 19 and Figure 20 show horizontal cross-sections for the liquid fraction field and their relative error. Both methods predict a very similar liquid fraction profile, with the liquid region remaining close to $f_{L}=1$ before rapidly decreasing toward $f_{L}=0$ at the solidification front. The location and overall shape of the liquid-to-solid transition are closely matched by both numerical approaches. The small differences can be observed in the mushy-zone region, where FVM results exhibit a slightly broader transition region, indicating a wider mushy zone than LRBFCM. Despite the differences, the results follow the same overall trends.

The maximum difference in liquid fraction reaches 0.15. This happens in the mushy-zone region near the solidification front. Outside these regions, the differences between liquid fraction fields in both numerical approaches remain below 0.05.

**Figure 21:**
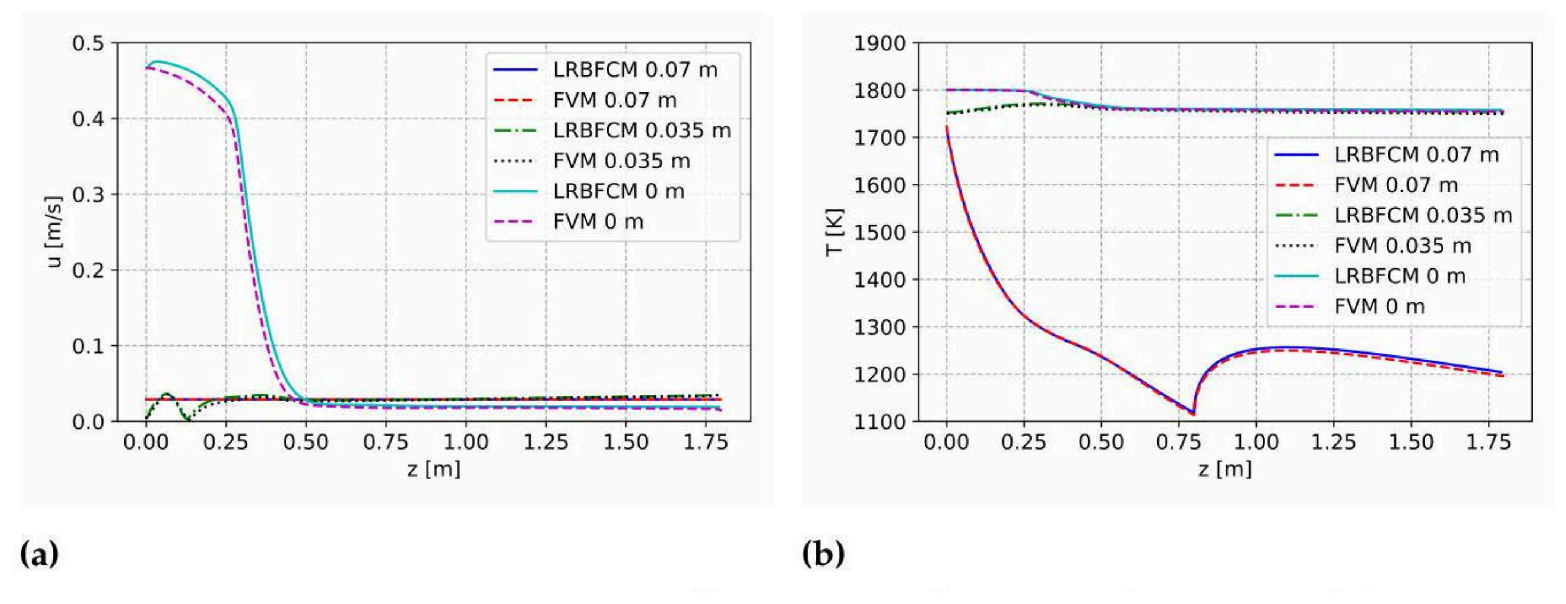
Comparison of LRBFCM and FVM results in the vertical direction. (**a**) Velocity field comparison at different cross-sections in the vertical direction; (**b**) Temperature field comparison at different cross-sections in the vertical direction.

**Figure 22:**
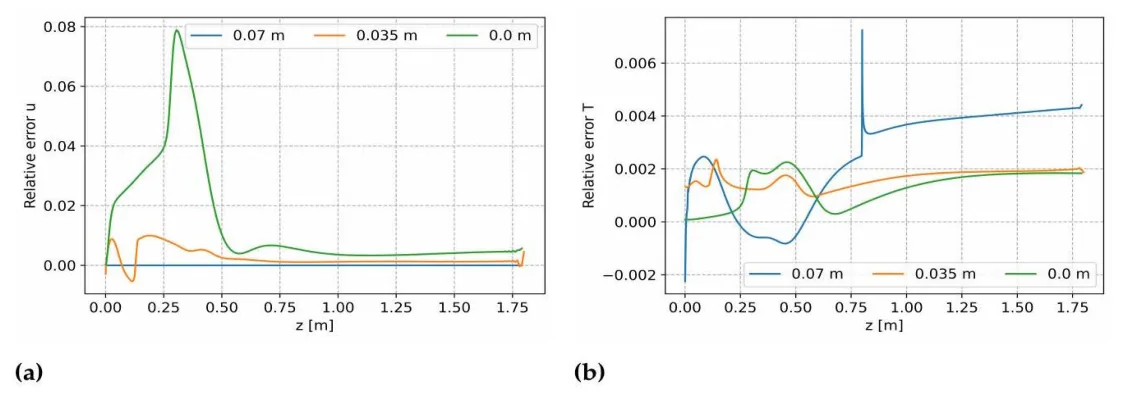
Relative error. (**a**) Relative error for velocity cross-sections in the vertical direction; (**b**) Relative error for temperature cross-sections in the vertical direction.

The vertical cross-sections are taken at radial distances r=0 m, which corresponds to the axis, r=0.035 m (which corresponds to the middle point between the axis and the outer wall), and at r=0.07 m (which coincides with the billet wall). Figure 21a shows vertical cross-sections of the velocity, and Figure 22a shows its error at the center and at the boundary of the billet and at the middle of the calculation domain. There is a difference in the central region, where the FVM results show lower velocities due to smaller recirculation zones than the LRBFCM results. The velocity at the wall is defined with a boundary condition and is not calculated. The temperature vertical cross-sections are shown in Figure 21b and their relative error in Figure 22b. The match between FVM and LRBFCM results at the boundary is very good. There is a difference between the results at the billet’s center and at the center of the calculation domain. The velocity at the center of the billet is slightly lower with the FVM approach in the mold region, whereas the velocities match in the spray-cooling region. In the center of the computational domain, the velocity is nearly identical between the two approaches. The temperature is lower for the FVM approach at both comparison cross-sections.

Overall, these results show that local discrepancies exist primarily in the upper mold region and in the mushy zone. The two approaches produce closely matching velocity fields and reasonably consistent temperature distributions over most of the domain, with the largest deviations confined to regions of steep thermal and phase-change gradients, which is somewhat expected.

**Figure 23:**
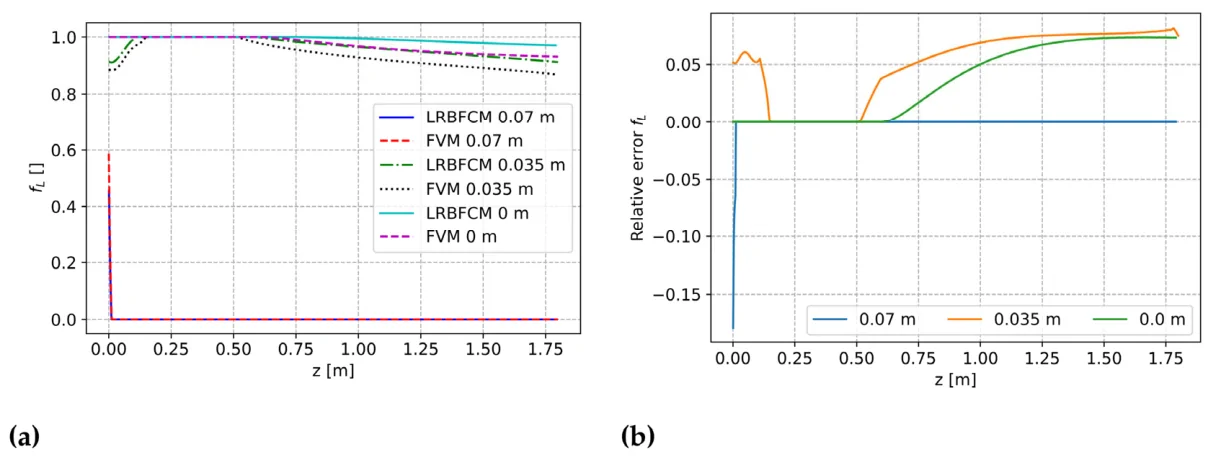
(**a**) Comparison of LRBFCM and FVM results in the vertical direction for liquid fraction; (**b**) Relative error for liquid fraction cross-sections in the vertical direction.

Figure 23 compares LRBFCM and FVM results for three vertical cross-sections and their relative errors. Overall, the agreement between the results calculated with both numerical methods is good. The small differences are evident in the mushy zone, where the FVM again yields lower values, indicating a slightly wider mushy zone. Overall, the results indicate that the liquid-fraction profiles accurately capture the solidification progression from the fully liquid top region through the intermediate solidification zone to the fully solidified region.

Both methods solve the same governing equations under the same boundary conditions and physical parameters. The main distinction between our in-house-developed LRBFCM simulation system and the FVM solution lies in the numerical formulation; the LRBFCM employs the strong (differential) form, whereas the FVM is based on a weak (integral) formulation over control volumes. Thus, the corresponding approximation or shape functions differ between the two methods, reflecting their different mathematical and numerical formulations rather than any difference in the underlying physical model. Furthermore, even under nominally identical physical and numerical settings, small differences in solution fields and derived quantities are expected, reflecting the inherent characteristics of each discretization approach rather than implementation inconsistencies. We previously tested the applicability of our method against analytical results as well as several benchmarks [36,37], yielding good results. However, these benchmarks are simple, with less extensive coupling between various fields.

A limitation of the present computational model is its restriction to r−z plane and its assumed axisymmetry. Consequently, the model cannot inherently represent 3D flow structures or possible symmetry-breaking instabilities, such as those discussed by [38]. The relevance of 3D effects may be particularly pertinent to CC of round billets, where nominally axisymmetric configurations can nevertheless exhibit asymmetric or biased flow under certain operating conditions. For example, asymmetric bias flow has been reported in round-billet molds subjected to mold electromagnetic stirring [39], with increasing stirring intensity leading to stronger deviations from the nominally symmetric flow pattern. Such 3D effects could potentially influence the local heat and mass transfer and, consequently, solidification behavior. Their assessment would require a fully 3D formulation, which is beyond the scope of the present study.

## 6. Conclusions

The detailed representation of the results in the paper expands the continuous casting benchmark in [40] and extends the one in [11]. While axisymmetry was already considered in [11], the present study extends that work in several important aspects. The simplified material properties and geometry enable comparison and verification with other numerical methods.

The results show good agreement between our in-house-developed LRBFCM and FVM. The differences between the methods remained within approximately 0.03 m/s in velocity and 15 K in temperature. Relative to the characteristic ranges encountered in continuous casting simulations, these deviations are small and indicate that both approaches consistently reproduce the main physical trends. From a technological standpoint, such differences are unlikely to significantly affect qualitative predictions of flow structure or global heat transfer. Due to the lack of experimental data, we cannot claim which results better approximate the problem’s solution. The comparison, therefore, primarily demonstrates consistency and mutual verification between independent numerical approaches.

The stability study showed that both the von Neumann stability condition and the CFL criterion must be satisfied to ensure stable simulations. When the discretization parameters and time step remained within the limits imposed by these criteria, the numerical solution was stable. Near or above these limits, the solution became conditionally stable and, in some cases, diverged or produced inaccurate results, highlighting the importance of meeting both criteria in practical computations.

In the future, the models will be upgraded with additional physics [41], including species transport, advanced solidification models [42], and the application of electromagnetic fields.

## Figures and Tables

**Table 1:** Billet computational domain dimensions.

Property	Notation	Value	Unit
Billet radius	$l_{R}$	0.07	m
Billet length	$l_{b}$	1.8	m
Mold length	$l_{m}$	0.8	m
Inlet radius	$r_{\mathrm{in}}$	0.0175	m
Wall length	$l_{\mathrm{wall}}$	0.1	m

**Table 2:** Temperature-independent material properties.

Property	Notation	Value	Unit
Density	ρ	7200	$kg/m^{3}$
Viscosity	μ	0.006	Pa· s
Specific heat	$c_{p}$	700	J/(kg·K)
Solidus temperature	$T_{S}$	1680	K
Liquidus temperature	$T_{L}$	1760	K
Melting enthalpy	$h_{m}$	250,000	J/kg
Thermal expansion coefficient	$\beta_{T}$	0.0001	1/K
Thermal conductivity	λ	30	W/(m·K)
Permeability coefficient	$K_{0}$	${1.6\cdot 10}^{-8}$	$1/m^{2}$

**Table 3:** Process parameters.

Property	Notation	Value	Unit
Casting velocity	$u_{\mathrm{cast}}$	1.75	m/min
Casting temperature	$T_{\mathrm{cast}}$	1800	K
Ambient temperature	$T_{0}$	293.15	K
Ambient pressure	$p_{0}$	0	Pa
Mold HTC	$h_{HTC,m}$	2000	$W/\left(m^{2}\cdot \;K\right)$
Secondary cooling zone HTC	$h_{HTC,sc}$	800	$W/\left(m^{2}\cdot \;K\right)$

**Table 4:** Number of nodes (In= Inlet, Mn= Meniscus, Mld = Mold, Spr = Spray, and All) and calculation times.

Name	In	Mn	Mld	Spr	All	Wall-Clock Time
S	10	30	41	45	3563	4 h 42 min
M	20	60	81	90	13,927	1 day 18 h 13 min
L	30	90	121	135	31,093	3 days 8 h 10 min
XL	40	120	161	180	55,058	7 days 11 h 22 min
XL_GRAD	40	120	161	180	55,058	6 days 22 h 57 min
XL_SEN	40	120	161	180	66,336	10 days 13 h 23 min
XXL	50	150	201	225	85,825	12 days 6 min
XXXL	60	180	241	270	123,388	22 days 1 h 11 min

**Table 5:** Time step convergence test.

Name	Time Step [s]	Writing Step [×1000]	Wall-Clock Time
$dt_{1}$	$2\times 10^{-3}$	0.5	2 min
$dt_{2}$	$10^{-3}$	1	3 min
$dt_{3}$	$5\times 10^{-4}$	2	7 min
$dt_{4}$	$2\times 10^{-4}$	5	15 min
$dt_{5}$	$10^{-4}$	10	29 min
$dt_{6}$	$5\times 10^{-5}$	20	1 h
$dt_{7}$	$2\times 10^{-5}$	50	2 h 29 min
$dt_{8}$	$10^{-5}$	100	4 h 42 min
$dt_{9}$	$5\times 10^{-6}$	200	12 h 23 min
$dt_{10}$	$2\times 10^{-6}$	500	1 day 6 h 23 min
$dt_{11}$	$10^{-6}$	1000	1 day 23 h 33 min
$dt_{12}$	$10^{-7}$	10,000	19 days 7 h 40 min

**Table 6:** Grid convergence study over 6 grids. $L_{st}$ represents the solid-shell thickness, $\mu_{t_{RMS}}$ is the global domain-averaged RMS value of $\mu_{t},$ $T_{RMS}$ is the global domain-averaged RMS value of T, and $\phi_{\mathrm{ex}.\;};\phi =L_{\mathrm{st}\;},\mu_{t_{RMS}},T_{\mathrm{RMS}}$ their extrapolated values. $N_{\mathrm{All}}$ is the number of nodes and $GCI_{\phi }$ is the grid convergence index in percent.

	XXXL	XXL	XL	XXL	XL	L
$N_{\mathrm{All}\;}$	123,388	85,823	55,058	85,823	55,058	31,093
$L_{st}\times 10^{-2}\;[m]$	1.214	1.213	1.220	1.213	1.220	1.208
$GCI_{L_{st}}$ %	0.17	3.03	-	1.28	1.63	-
$L_{st,ex.}\times 10^{-2}\;[m]$		1.21			1.20	
$\mu_{t_{RMS}}\times 10^{-5}\;[Pa/s]$	4.698	4.685	4.663	4.685	4.663	4.618
${GCI}_{\mu_{T_{RMS}}}$ %	2.39	3.23	-	3.14	4.58	-
$\mu_{t_{RMS},ex}\times 10^{-5}\;[Pa/s]$		4.74			4.73	
$T_{\mathrm{RMS}\;}\;[K]$	1643	1643	1642	1643	1642	1642
$GCI_{T_{\mathrm{RMS}\;}}\;\%$	0.11	0.17	-	0.79	0.85	-
$T_{\mathrm{RMS}\;,\;\mathrm{ex}.}$ [K]		1643			1648	
	XL	L	M	L	M	S
$N_{\mathrm{All}\;}$	55,058	31,093	13,927	31,093	13,927	3563
$L_{st}\times 10^{-2}\;[m]$	1.220	1.208	1.193	1.208	1.193	1.227
$GCI_{L_{st}}$ %	19.3	17.29	-	4.32	4.51	-
$L_{st,ex.}\times 10^{-2}\;[m]$		1.32			1.23	
$\mu_{t_{RMS}}\times 10^{-5}\;[Pa/s]$	4.663	4.618	4.519	4.618	4.519	4.206
${GCI}_{\mu_{T_{RMS}}}$ %	5.76	8.57	-	9.78	15.16	-
$\mu_{t_{RMS},ex}\times 10^{-5}\;[Pa/s]$		4.75			4.77	
$T_{\mathrm{RMS}}\;[K]$	1642	1642	1640	1642	1640	1636
$GCI_{T_{\mathrm{RMS}}}\;\%$	0.40	0.53	-	0.44	0.63	-
$T_{\mathrm{RMS}\;,\;\mathrm{ex}.}$ [K]		1644			1644	

**Table 7:** Temporal convergence study over 12 time steps. $L_{st}$ represents the solid-shell thickness, $\mu_{t_{RMS}}$ is the global domain-averaged RMS value of $\mu_{t},T_{RMS}$ is the global domain-averaged RMS value of T, and $\phi_{\mathrm{ex}.\;};\phi =L_{st},\mu_{t_{\mathrm{RMS}\;}},T_{\mathrm{RMS}\;}$ their extrapolated values. Δt is the time step and $TCI_{\phi }$ is the grid convergence index in percent.

	$dt_{1}$	$dt_{2}$	$dt_{3}$	$dt_{2}$	$dt_{3}$	$dt_{4}$	$dt_{3}$	$dt_{4}$	$dt_{5}$
Δ $t\times 10^{-4}\;[s]$	20	10	5	10	5	2	5	2	1
$L_{st}\times 10^{-2}\;[m]$	1.2282	1.2281	1.2280	1.2281	1.2280	1.2279	1.2280	1.2279	1.2276
$TCI_{L_{st}}$ %	0.17	3.03	-	1.28	1.63	-	19.3	17.29	-
$L_{\mathrm{st},\mathrm{ex}.\;}\times 10^{-2}\;[m]$		1.2278			1.2279			1.2276	
$\mu_{t_{\mathrm{RMS}\;}}\times 10^{-5}\;[Pa/s]$	4.10	4.12	4.13	4.12	4.13	4.15	4.13	4.15	4.19
$TCI_{\mu_{t_{RMS}}}\times 10^{-2}$%	4.97	5.44	-	4.40	4.96	-	0.451	0.134	-
$\mu_{t_{\mathrm{RMS},\mathrm{ex}.\;}}\times 10^{-5}\;[Pa/s]$		4.30			4.30			20	
$T_{\mathrm{RMS}}\;[K]$	1636.4	1636.4	1636.4	1636.4	1636.4	1636.3	1636.4	1636.3	1636.2
$TCI_{T_{\mathrm{RMS}\;}}\times 10^{-4}\%$	4.30	6.03	-	1.86	3.26	-	0.499	0.0194	-
$T_{\mathrm{RMS}\;,\;\mathrm{ex}.\;}$ [K]		1634.5			1634.4			1635.8	
	$dt_{4}$	$dt_{5}$	$dt_{6}$	$dt_{5}$	$dt_{6}$	$dt_{7}$	$dt_{6}$	$dt_{7}$	$dt_{8}$
$\Delta t\times 10^{-5}\;[s]$	2	1	0.5	1	0.5	0.2	0.5	0.2	0.1
$L_{st}\times 10^{-2}\;[m]$	1.2279	1.2276	1.2275	1.2276	1.2275	1.2275	1.2275	1.2275	1.2275
$TCI_{L_{st}}$ %	0.17	4.32	4.51	-	3.03	-	1.28	1.63	-
$L_{st,ex.}\times 10^{-2}\;[m]$		1.2275			1.2275			1.2278	
$\mu_{t_{\mathrm{RMS}\;}}\times 10^{-5}\;[Pa/s]$	4.15	4.19	4.20	4.19	4.20	4.20	4.20	4.20	4.21
$TCI_{\mu_{t_{RMS}}}\cdot 10^{-3}$%	2.17	6.05	-	5.10	2.06	-	1.47	2.13	-
$\mu_{t_{\mathrm{RMS},\mathrm{ex}.\;}}\times 10^{-5}\;[Pa/s]$		4.21			4.21			4.21	
$T_{\mathrm{RMS}}\;[K]$	1636.3	1636.2	1636.1	1636.2	1636.1	1636.0	1636.1	1636.0	1635.7
$TCI_{T_{\mathrm{RMS}\;}}\times 10^{-4}\%$	0.454	2.61	-	0.202	0.960	-	1.63	1.10	-
$T_{\mathrm{RMS}\;,\;\mathrm{ex}.\;}$ [K]		1636.2			1636.2			1636.4	
	$dt_{7}$	$dt_{8}$	$dt_{9}$	$dt_{8}$	$dt_{9}$	$dt_{10}$	$dt_{9}$	$dt_{10}$	$dt_{11}$
$\Delta t\times 10^{-6}\;[s]$	2	1	0.5	1	0.5	0.2	0.5	0.2	0.1
$L_{st}\times 10^{-2}\;[m]$	1.2275	1.2275	1.2275	1.2275	1.2275	1.2275	1.2275	1.2275	1.2275
$TCI_{L_{st}}$ %	0.17	19.3	17.29	-	4.32	4.51	-	3.03	-
$L_{st,ex.}\times 10^{-2}\;[m]$		1.2273			1.2275			1.2275	
$\mu_{t_{\mathrm{RMS}\;}}\times 10^{-5}\;[Pa/s]$	4.20	4.21	4.21	4.21	4.21	4.21	4.21	4.21	4.21
$TCI_{\mu_{t_{RMS}}}\times 10^{-4}$%	2.21	5.34	-	7.01	12.1	-	5.59	3.83	-
$\mu_{t_{\mathrm{RMS},\mathrm{ex}.\;}}\times 10^{-5}\;[Pa/s]$		4.21			4.20			4.20	
$T_{\mathrm{RMS}}\;[K]$	1636.0	1635.7	1634.1	1635.7	1634.1	1633.9	1634.1	1633.9	1633.7
$TCI_{T_{\mathrm{RMS}\;}}\times 10^{-4}\%$	6.06	6.77	-	0.158	0.505	-	0.0411	0.115	-
$T_{\mathrm{RMS}\;,\;\mathrm{ex}.\;}\;[K]$		1637.1			1636.4			1636.4	
	$dt_{10}$	$dt_{11}$	$dt_{12}$						
$\Delta t\times 10^{-6}\;[s]$	2	1	0.1						
$L_{st}\times 10^{-2}\;[m]$	1.2275	1.2275	1.2275						
$TCI_{L_{st}}\;\%$	1.28	1.63	-						
$L_{st,ex.}\times 10^{-2}\;[m]$		1.2275							
$\mu_{t_{\mathrm{RMS}\;}}\times 10^{-5}\;[Pa/s]$	4.21	4.21	4.20						
$TCI_{\mu_{t_{RMS}}}\times 10^{-4}\%$	3.38	8.29	-						
$\mu_{t_{\mathrm{RMS},\mathrm{ex}.\;}}\times 10^{-5}\;[Pa/s]$		4.20							
$T_{\mathrm{RMS}}\;[K]$	1633.9	1633.7	1633.4						
$TCI_{T_{\mathrm{RMS}\;}}10^{-6}\%$	0.00224	1.31	-						
$T_{\mathrm{RMS}\;,\;\mathrm{ex}.\;}$ [K]		1636.4							

**Table 8:** Minimum distance between nodes in radial r and axial z directions.

Name	Δr [m]	Δz [m]	Δr/Δz
S	$4.41998\times 10^{-4}$	$5.0788\times 10^{-3}$	0.087
M	$2.21998\times 10^{-4}$	$2.5496\times 10^{-3}$	0.087
L	$1.48997\times 10^{-4}$	$1.7000\times 10^{-3}$	0.087
XL	$1.11997\times 10^{-4}$	$1.2771\times 10^{-3}$	0.087
XXL	$8.8997\times 10^{-5}$	$1.0200\times 10^{-3}$	0.087
XXXL	$7.3999\times 10^{-5}$	$8.5002\times 10^{-4}$	0.087

**Table 9:** Von Neumann stability criteria in the radial Δr direction. Bold: calculated cases diverge.

Nodes	S	M	L	XL	XXL	XXXL
Δt [s]						
$2\times 10^{-3}$	$6.09\times 10^{-2}$	$2.42\times 10^{-1}$	$5.36\times 10^{-1}$	$9.49\times 10^{-1}$	**1.50**	**2.17**
$10^{-3}$	$3.05\times 10^{-2}$	$1.21\times 10^{-1}$	$2.68\times 10^{-1}$	$4.75\times 10^{-1}$	$7.52\times 10^{-1}$	**1.09**
$5\times 10^{-4}$	$1.52\times 10^{-2}$	$6.04\times 10^{-2}$	$1.34\times 10^{-1}$	$2.37\times 10^{-1}$	$3.76\times 10^{-1}$	$5.44\times 10^{-1}$
$2\times 10^{-4}$	$6.09\times 10^{-3}$	$2.42\times 10^{-2}$	$5.36\times 10^{-2}$	$9.49\times 10^{-2}$	$1.50\times 10^{-1}$	$2.17\times 10^{-1}$
$10^{-4}$	$3.05\times 10^{-3}$	$1.21\times 10^{-2}$	$2.68\times 10^{-2}$	$4.75\times 10^{-2}$	$7.52\times 10^{-2}$	$1.09\times 10^{-1}$
$5\times 10^{-5}$	$1.52\times 10^{-3}$	$6.04\times 10^{-3}$	$1.34\times 10^{-2}$	$2.37\times 10^{-2}$	$3.76\times 10^{-2}$	$5.44\times 10^{-2}$
$2\times 10^{-5}$	$6.09\times 10^{-4}$	$2.42\times 10^{-3}$	$5.36\times 10^{-3}$	$9.49\times 10^{-3}$	$1.50\times 10^{-2}$	$2.17\times 10^{-2}$
$10^{-5}$	$3.05\times 10^{-4}$	$1.21\times 10^{-3}$	$2.68\times 10^{-3}$	$4.75\times 10^{-3}$	$7.52\times 10^{-3}$	$1.09\times 10^{-2}$
$5\times 10^{-6}$	$1.52\times 10^{-4}$	$6.04\times 10^{-4}$	$1.34\times 10^{-3}$	$2.37\times 10^{-3}$	$3.76\times 10^{-3}$	$5.44\times 10^{-3}$
$2\times 10^{-6}$	$6.09\times 10^{-5}$	$2.42\times 10^{-4}$	$5.36\times 10^{-4}$	$9.49\times 10^{-4}$	$1.50\times 10^{-3}$	$2.17\times 10^{-2}$
$10^{-6}$	$1.52\times 10^{-4}$	$1.21\times 10^{-4}$	$2.68\times 10^{-4}$	$4.75\times 10^{-4}$	$7.52\times 10^{-4}$	$1.09\times 10^{-4}$
$10^{-7}$	$1.52\times 10^{-5}$	$1.21\times 10^{-5}$	$2.68\times 10^{-5}$	$4.75\times 10^{-5}$	$7.52\times 10^{-5}$	$1.09\times 10^{-4}$

**Table 10:** Von Neumann stability criteria in the axial Δz direction. Bold: calculated cases diverge.

Nodes	S	M	L	XL	XXL	XXXL
Δt [s]						
$2\times 10^{-3}$	$4.62\times 10^{-4}$	$1.83\times 10^{-4}$	$4.12\times 10^{-3}$	$7.30\times 10^{-3}$	$1.14\times 10^{-2}$	$1.65\times 10^{-2}$
$10^{-3}$	$2.31\times 10^{-4}$	$9.16\times 10^{-4}$	$2.06\times 10^{-3}$	$3.65\times 10^{-3}$	$5.72\times 10^{-3}$	$8.24\times 10^{-3}$
$5\times 10^{-4}$	$1.15\times 10^{-4}$	$4.58\times 10^{-4}$	$1.03\times 10^{-3}$	$1.82\times 10^{-3}$	$2.86\times 10^{-3}$	$4.12\times 10^{-3}$
$2\times 10^{-4}$	$4.62\times 10^{-5}$	$1.83\times 10^{-5}$	$4.12\times 10^{-4}$	$7.30\times 10^{-4}$	$1.14\times 10^{-3}$	$1.65\times 10^{-3}$
$10^{-4}$	$2.31\times 10^{-5}$	$9.16\times 10^{-5}$	$2.06\times 10^{-4}$	$3.65\times 10^{-4}$	$5.72\times 10^{-4}$	$8.24\times 10^{-4}$
$5\times 10^{-5}$	$1.15\times 10^{-5}$	$4.58\times 10^{-5}$	$1.03\times 10^{-4}$	$1.82\times 10^{-4}$	$2.86\times 10^{-4}$	$4.12\times 10^{-4}$
$2\times 10^{-5}$	$4.62\times 10^{-6}$	$1.83\times 10^{-6}$	$4.12\times 10^{-5}$	$7.30\times 10^{-5}$	$1.14\times 10^{-4}$	$1.65\times 10^{-4}$
$10^{-5}$	$2.31\times 10^{-6}$	$9.16\times 10^{-6}$	$2.06\times 10^{-5}$	$3.65\times 10^{-5}$	$5.72\times 10^{-5}$	$8.24\times 10^{-5}$
$5\times 10^{-6}$	$1.15\times 10^{-6}$	$4.58\times 10^{-6}$	$1.03\times 10^{-5}$	$1.82\times 10^{-5}$	$2.86\times 10^{-5}$	$4.12\times 10^{-5}$
$2\times 10^{-6}$	$4.62\times 10^{-7}$	$1.83\times 10^{-7}$	$4.12\times 10^{-6}$	$7.30\times 10^{-6}$	$1.14\times 10^{-5}$	$1.65\times 10^{-5}$
$10^{-6}$	$2.31\times 10^{-7}$	$9.16\times 10^{-7}$	$2.06\times 10^{-6}$	$3.65\times 10^{-6}$	$5.72\times 10^{-6}$	$8.24\times 10^{-6}$
$10^{-7}$	$2.31\times 10^{-8}$	$9.16\times 10^{-8}$	$2.06\times 10^{-7}$	$3.65\times 10^{-7}$	$5.72\times 10^{-7}$	$8.24\times 10^{-7}$

**Table 11:** CFL criterion results. Italics: calculated cases diverge. Bold: calculated cases converge and do not fit the CFL criterion. Other: calculated cases converge and fit the CFL criterion.

Nodes	S	M	L	XL	XXL	XXXL
Δt [s]						
$2\times 10^{-3}$	**2.27**	*8.97*	*20.18*	*35.77*	*56.07*	*81.50*
$10^{-3}$	**1.13**	*4.49*	*10.09*	*17.88*	*28.03*	*40.75*
$5\times 10^{-4}$	$5.66\times 10^{-1}$	**2.24**	*5.05*	*8.94*	*14.02*	*20.37*
$2\times 10^{-4}$	$2.27\times 10^{-1}$	$8.97\times 10^{-1}$	**2.02**	*3.58*	*5.61*	*8.15*
$10^{-4}$	$1.13\times 10^{-1}$	$4.49\times 10^{-1}$	**1.00**	**1.79**	**2.80**	**4.08**
$5\times 10^{-5}$	$5.66\times 10^{-2}$	$2.24\times 10^{-1}$	$5.05\times 10^{-1}$	$8.94\times 10^{-1}$	**1.40**	**2.04**
$2\times 10^{-5}$	$2.27\times 10^{-2}$	$8.97\times 10^{-2}$	$2.02\times 10^{-1}$	$3.58\times 10^{-1}$	$5.61\times 10^{-1}$	$8.15\times 10^{-1}$
$10^{-5}$	$1.13\times 10^{-2}$	$4.49\times 10^{-2}$	$1.00\times 10^{-1}$	$1.79\times 10^{-1}$	$2.80\times 10^{-1}$	$4.08\times 10^{-1}$
$5\times 10^{-6}$	$5.66\times 10^{-3}$	$2.24\times 10^{-2}$	$5.05\times 10^{-2}$	$8.94\times 10^{-2}$	0.14	0.204
$2\times 10^{-6}$	$2.27\times 10^{-3}$	$8.97\times 10^{-3}$	$2.02\times 10^{-2}$	$3.58\times 10^{-2}$	$5.61\times 10^{-2}$	$8.15\times 10^{-2}$
$10^{-6}$	$1.13\times 10^{-3}$	$4.49\times 10^{-3}$	$1.00\times 10^{-2}$	$1.79\times 10^{-2}$	$2.80\times 10^{-2}$	$4.08\times 10^{-2}$
$10^{-7}$	$1.13\times 10^{-4}$	$4.49\times 10^{-4}$	$1.00\times 10^{-3}$	$1.79\times 10^{-3}$	$2.80\times 10^{-3}$	$4.08\times 10^{-3}$

## Data Availability

The data presented in this study are available on request from the corresponding author due to proprietary industrial information and a non-disclosure agreement (NDA) with the industrial partner, Štore Steel d.o.o. Requests for access to selected datasets may be considered on a case-by-case basis, subject to review and approval by the industrial partner.

## Abbreviations

The following abbreviations are used in this manuscript:

LRBFCM	Local Radial Basis Function Collocation Method
FVM	Finite Volume Method
CC	Continuous casting
3D	Three-dimensional
FDM	Finite difference method
RANS	Reynolds-Averaged Navier–Stokes
LES	Large eddy simulation
DNS	Direct numerical simulation
SEN	Submerged entry nozzle
HTC	Heat transfer coefficient
CFL	Courant-Friedrichs-Levy
FSM	Fractional step method
FPM	Finite Point Method
2D	Two-dimensional
